# Review of advanced sensor system applications in grinding operations

**DOI:** 10.1016/j.jare.2025.01.013

**Published:** 2025-01-10

**Authors:** Danil Yu. Pimenov, Leonardo Rosa Ribeiro da Silva, Mustafa Kuntoğlu, Bruno Souza Abrão, Luiz Eduardo dos Santos Paes, Emanoil Linul

**Affiliations:** a Department of Automated Mechanical Engineering, South Ural State University, Lenin Prosp. 76, Chelyabinsk 454080 Russia; b Faculty of Mechanical Engineering, Federal University of Uberlândia, Av. João Naves de Ávila, 2121, 38400-902, Uberlândia, MG, Brazil; c Selcuk University, Technology Faculty, Mechanical Engineering Department, Konya 42130, Turkey; d Department of Mechanics and Strength of Materials, Politehnica University Timisoara, 1 Mihai Viteazu Avenue, 300 222 Timisoara, Romania

**Keywords:** Grinding, Machining, Sensor systems, Tool condition monitoring, Conventional and artificial intelligence applications

## Abstract

**Background:**

Today, in a wide variety of industries, grinding operations are an extremely important finishing process for obtaining precise dimensions and meeting strict requirements for roughness and shape accuracy. However, the constant wear of abrasive tools during grinding negatively affects the dimensional and surface conditions of the workpiece. Therefore, effective monitoring of the wear process during grinding operations helps to predict tool life, plan maintenance and ensure consistent product quality.

**Aim of Review:**

The objective of this review is to examine current tool condition monitoring techniques, both direct and indirect, in various sensor systems and their application in both traditional and AI-driven grinding processes. By examining these techniques, the review provides insight into how different monitoring techniques can improve process efficiency, reduce downtime, and improve finished product quality, as well as the application of intelligent and adaptive processes to traditional grinding operations.

**Key Scientific Concepts of Review:**

The review discusses the critical role of sensor systems in monitoring tool condition, including technologies such as imaging, vibration analysis, acoustic emission, and force measurement. These systems are vital for detecting wear and predicting failures, allowing for timely interventions and preventing unplanned downtimes. The integration of artificial intelligence into these monitoring systems greatly enhances their capabilities, as they enable more proactive strategies and adapt to changing conditions during the grinding process.

## Highlights

• Comprehensive review of sensor systems in grinding operations.
• Evaluation of AI and conventional tool condition monitoring.
• Analysis of sensor applications for in-line process monitoring.
• Discussion on temperature, force, and surface roughness measurements.
• Exploration of Industry 4.0 integration in grinding processes.

## Graphical abstract

**Figure:**
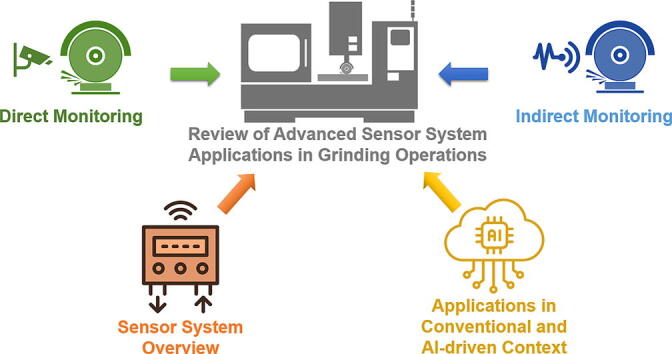

## Introduction

The grinding process (GP) is an operation in manufacturing usually employed to achieve precise dimensions and meeting stringent requirements for surface roughness and shape accuracy. This process is particularly crucial in industries such as automotive, aerospace, and tool manufacturing, where the quality of the final product is paramount. GPs are used for finishing machining with an abrasive tool, with the primary objective of achieving the specified surface roughness [1] and ensuring the workpiece tolerances [2]. This process is significantly influenced by the wear that cause changes in abrasive wheel geometry [3].

Pereverzev and Pimenov [4] demonstrated that as the abrasive grains wear down, cutting forces increase, leading to elastic deflections of the abrasive tool from the part. These changes impact micro and macro tolerances of the machined part [5]. Additionally, excessive wear can alter the geometric shape of the grinding wheels (GWs) due to intense chipping and grain separation, potentially resulting in tool breakage [6]. Based on that, monitoring the abrasive tool condition in the grinding is crucial for maintaining the desired surface roughness, geometric shape, and dimensional accuracy [7].

Currently, both direct and indirect monitoring methods are commonly used for various machining processes [8]. Direct monitoring methods involve sensory systems that evaluate the tool condition by directly observing the cutting part of the tool. As noted by Rehorn et al. [9], direct measurement of wear parameters involves monitoring front surface wear, flank wear, tool volume or weight reduction, and cutting edge wear. These can be measured using optical, laser, electromechanical, ultrasonic, or pneumatic methods.

Indirect monitoring methods use sensors to infer tool condition by tracking parameters such as force, vibration, acoustic emission (AE), current, power, and imaging during the machining process [10]. Despite the availability of numerous review articles that discuss sensor systems for turning [11], for milling [12], for drilling [13], for grinding [14], [15], or a combination of different machining types [16], there is still a lack of comprehensive studies on sensor systems specifically for grinding operations, particularly those incorporating artificial intelligence (AI) [17].

The integration of AI into sensor systems has revolutionized monitoring and data analysis in machining processes. AI-driven sensors outperform traditional sensors by offering superior accuracy, faster response times, and greater adaptability to complex machining environments [18]. Leveraging advanced machine learning (ML) algorithms, these sensors process and analyze data in real time, significantly enhancing performance metrics. For example, studies indicate that AI-driven sensors deliver up to 35% higher accuracy in detecting anomalies and predicting system failures compared to traditional sensors, which rely on fixed algorithms or manual calibration [19]. Furthermore, they demonstrate a 40% faster response time in dynamic environments, such as industrial automation and autonomous vehicles, where rapid decision-making is essential [20]. Another key advantage is their adaptability; AI-driven sensors learn and adjust to complex, multi-variable conditions, reducing error rates by 25% in scenarios where traditional sensors struggle with static programming [21]. These advancements underscore the growing preference for AI-driven sensors in advanced industrial and technological applications [22].

Advanced sensor system applications in grinding operations play a pivotal role in improving the sustainability of the process by enabling real-time monitoring, optimizing resource utilization, and reducing waste. Sensors such as force, vibration, AE, and temperature sensors provide critical data on the GWs condition, workpiece surface quality, and overall process stability [23]. By leveraging this data, manufacturers can implement predictive maintenance strategies to minimize tool wear and prevent unexpected failures, reducing the need for frequent tool replacements and extending the GWs lifespan. Furthermore, advanced sensor systems combined with AI enable adaptive control of grinding parameters, such as feed rate, cutting speed, and depth of cut, ensuring optimal material removal rates (MRRs) with minimal energy consumption. This not only reduces the environmental footprint of the process but also enhances the efficiency and precision of grinding operations [24].

In addition to improving process efficiency, sensor systems contribute to sustainability by minimizing material waste and rework. Real-time monitoring allows for early detection of process anomalies, such as excessive vibrations, thermal damage, or tool wear, which can lead to defective parts. By addressing these issues promptly, manufacturers can reduce the production of scrap materials and avoid unnecessary energy expenditure on reprocessing [25]. Moreover, sensors that monitor coolant flow and temperature help optimize the use of cutting fluids, reducing both consumption and disposal costs, which are key concerns in sustainable manufacturing [26]. The integration of multi-sensor systems with IoT (Internet of Things) and AI technologies further enhances their capabilities by enabling remote monitoring and data-driven decision-making, making GPs more adaptive and environmentally friendly. Ultimately, advanced sensor systems not only improve the economic efficiency of grinding operations but also align them with the principles of sustainable manufacturing by reducing waste, conserving resources, and minimizing environmental impact [23].

This article examines key direct and indirect systems for grinding, such as sensors for force, vibration, AE, current, power, imaging, temperature, ultrasonic, optical, and laser detection. It also covers the use of AI in monitoring tool condition.

**Fig. 1:**
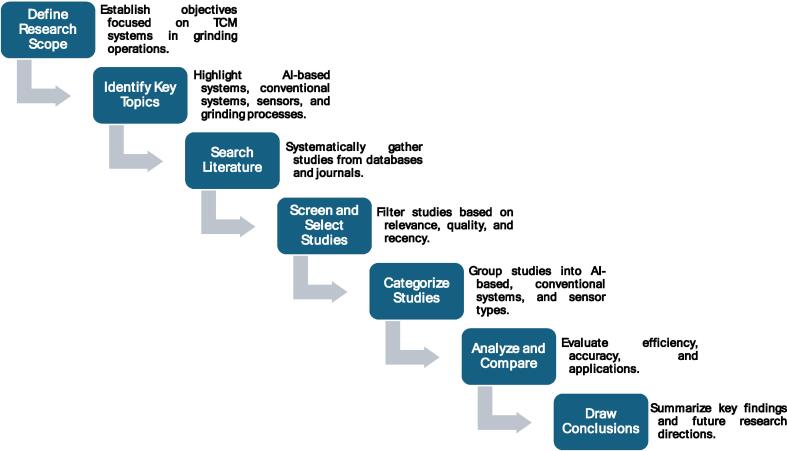
Overview of the review methodology.

The methodology of this paper is ilustrated by the flowchart represented in Fig. 1, that outlines the overall review methodology, offering a clear and systematic representation of the key stages, from defining the research scope to analyzing and synthesizing findings. Section 1 introduces the grinding process, emphasizing its importance in manufacturing for achieving high precision and superior surface quality. This section highlights the critical role grinding plays across various industries. Section 2 examines the influence of the grinding wheel's condition on the process, addressing topics such as grinding schemes, classification, and the relationship between wheel condition, grinding accuracy, and common defects. Section 3 focuses on sensor systems used for monitoring tool conditions in grinding, discussing both direct and indirect monitoring methods, with particular attention to the advancements brought by AI-powered sensors. Section 4 delves deeper into indirect monitoring techniques, providing an in-depth analysis of sensor types such as force, vibration, and acoustic emission sensors, and exploring their applications in both conventional and AI-driven systems. Section 5 identifies the challenges faced by current grinding sensor systems and explores future trends and potential advancements in the field. Finally, Section 6 concludes the paper by summarizing the evaluation of sensor systems for tool condition monitoring, comparing traditional and AI-enhanced approaches, and outlining their respective strengths and limitations.

The main purpose of the paper is to review sensor systems for monitoring the condition of grinding tools, focusing on both conventional and AI-enhanced methods. The research explores how different sensor technologies can improve the accuracy and efficiency of the GP by providing real-time monitoring and feedback. By examining the impact of GW conditions on process outcomes, the paper evaluates the advantages and disadvantages of various sensor systems, highlighting their potential to enhance precision and reduce defects in manufacturing. This comprehensive review addresses future trends and challenges, guiding advancements in grinding technology.

## Impact of the grinding wheel's condition on the grinding process

The condition of the GW is crucial in the GP, significantly impacting the final quality, roughness, and geometric tolerances of the manufactured part. The overall condition of the GW includes factors such as its geometry, the composition of the bonding material, and the size and distribution of the abrasive grains [27].

During grinding operations, the uniformity of material removal is directly influenced by the state of the GW. A GW with sharp abrasive grains ensures consistent material removal from the workpiece. Conversely, a worn GW can cause uneven material removal, affecting both the surface and subsurface integrity of the part [28].

Over time, the condition of the GW deteriorates due to wear mechanisms such as grain fracture, bond degradation, and loading, where material adheres to the GW surface [29]. These changes reduce the GW cutting capacity, leading to an increase in grinding forces and vibrations. This results not only in reduced MRRs but also in potential surface defects on the workpiece, such as chatter marks or uneven finishes [30]. Additionally, the GW ability to self-sharpen diminishes as abrasive grains become dull or clogged with debris, further compromising the GP [31]. Understanding these wear mechanisms and implementing timely dressing or truing operations is critical to restoring the GW cutting efficiency and ensuring consistent grinding outcomes.

Heat generation during grinding, influenced by the abrasive wheel's condition, is crucial. A worn GW increases friction, causing excessive heat that can lead to thermal damage, metallurgical changes, or distortions, negatively impacting the workpiece's quality and dimensional accuracy [32].

The efficiency of the GP is also impacted by the GW condition. A GW with the appropriate grain size and binder can facilitate efficient material removal, thereby reducing the time and energy required for the operation. Conversely, a GW in poor condition can increase process time, energy consumption, and wear [33]. As the GW deteriorates over time, its impact on processing efficiency and energy consumption becomes increasingly significant. A worn GW requires higher grinding forces to achieve material removal, which directly leads to increased energy consumption [34]. This inefficiency not only raises operational costs but also contributes to greater heat generation, which can negatively affect both the GW and the workpiece. Furthermore, the reduced cutting efficiency of a worn GW increases the time required to complete the GP, further decreasing overall productivity. Maintaining the GW in optimal condition is therefore critical to minimizing energy usage, improving processing efficiency, and ensuring consistent, high-quality results [35].

The GW condition can also influence other aspects in the workpiece, such as surface integrity and residual stresses. A poorly conditioned GW can cause subsurface damage and induce tensile residual stresses, compromising the mechanical properties and performance of the part as it could lead to increased wear or surface cracks [31].

Maintaining an ideal condition of the GW is essential to achieve efficient and high-quality grinding operations [36]. Regular inspection and preparation of the GW, appropriate selection of parameters such as grit size and binder material, as well as proper handling and storage can help preserve the GW condition, thus ensuring consistent grinding performance [37].

### Grinding processes and their classification

**Fig. 2:**
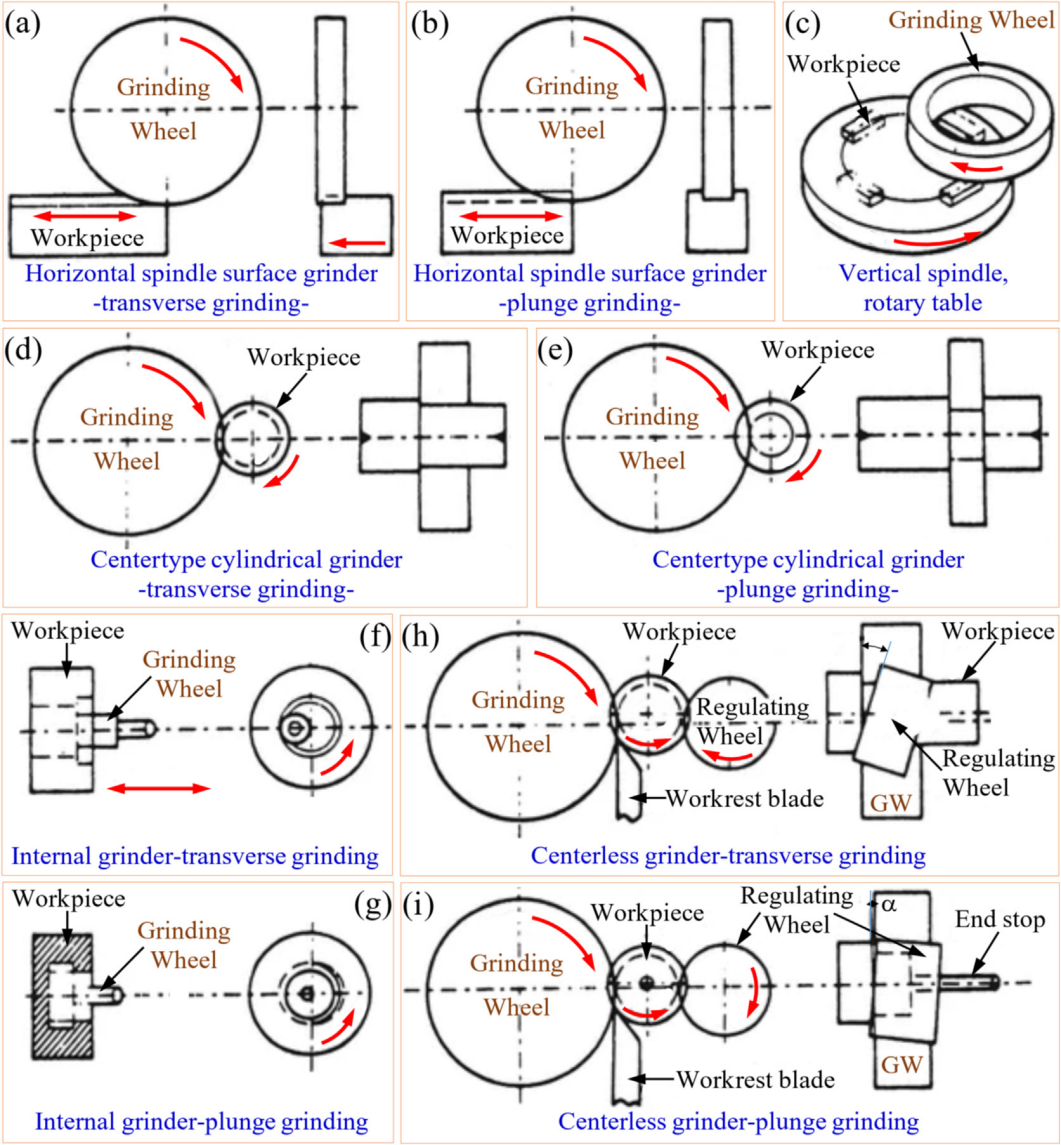
Grinding processes classification.

Adapted from Kalpakjian [39]

Grinding process is comprised of a wide range of methods and classifications adapted to achieve specific results on different cutting conditions. These methods have been refined over time to meet distinct requirements across various applications to achieve unique demands on precision, surface finish and productivity [38]. In Fig. 2 are shown the main types of GPs.

In horizontal spindle surface grinding (Fig. 2a), traverse grinding is one of the most common methods. The GW rotates on a horizontal spindle while the workpiece moves back and forth beneath it. The GW gradually removes material across the surface of the workpiece, making this method ideal for achieving flat surfaces with high precision. It is commonly used for machining large, flat components where uniform material removal is critical [40]. In contrast, plunge grinding with a horizontal spindle (Fig. 2b) involves the GW plunging directly into the workpiece without any lateral movement. This operation is suitable for localized material removal, such as creating grooves or specific surface profiles [41].

In vertical spindle surface grinding (Fig. 2c), rotary table grinding involves mounting the GW on a vertical spindle, and the workpiece is placed on a rotating table. The rotary motion of the table ensures even exposure of the workpiece to the GW, making this method ideal for achieving uniform finishes on circular or flat surfaces. This process is particularly effective for machining large workpieces, such as gear blanks or plates, where consistent surface quality is essential [42].

Centertype cylindrical grinding is another significant operation used for machining cylindrical components. In traverse grinding (Fig. 2d), the workpiece, supported at its ends, rotates while the GW moves along its longitudinal axis. This method is widely used for machining shafts, rods, and other cylindrical parts, as it produces precise diameters and smooth finishes [43]. Alternatively, in plunge grinding (Fig. 2e), the GW is fed radially into the rotating workpiece, removing material from a specific section. This method is commonly employed for machining features such as shoulders or grooves that require precise radial dimensions [44].

Internal grinding focuses on machining the internal surfaces of a workpiece, such as bores or holes. In internal traverse grinding (Fig. 2f), the GW rotates while the workpiece moves back and forth, ensuring even material removal. This method is ideal for achieving tight tolerances and smooth finishes in internal geometries [45]. Meanwhile, internal plunge grinding (Fig. 2g) involves feeding the GW radially into the internal surface of the workpiece, removing material from a specific area. This operation is often used for machining internal features like grooves, shoulders, or specific bore dimensions [46].

Finally, centerless grinding is a versatile operation for machining cylindrical parts without the need for centers to support the workpiece. In centerless traverse grinding (Fig. 2h), the workpiece is supported between a regulating wheel and a workrest blade while the GW removes material. The regulating wheel controls the workpiece's rotation and axial movement, while the GW performs the cutting. This method is efficient for machining cylindrical parts in high volumes, such as pins or rods, with consistent precision [47]. In centerless plunge grinding (Fig. 2i), the workpiece remains stationary in the axial direction while the GW feeds radially into it. This method is particularly useful for machining parts with stepped diameters or specific profiles. The regulating wheel ensures proper support and control during the operation [48].

Each of these grinding operations is tailored to specific machining needs, offering flexibility to achieve desired surface finishes, geometries, and tolerances. The choice of operation depends on factors such as the workpiece's geometry, the required precision, and the specific machining requirements.

### Assessing grinding wheel condition relation to grinding accuracy

As previously stated, GW condition significantly influences the GP, affecting crucial parameters such as dimensional accuracy, roughness and geometric shape deviations. These parameters are vital for evaluating the final part quality and performance in manufacturing processes [49].

Dimensional accuracy refers to the extent to which the dimensions of the part match the design specifications after the processing, and the GW condition can heavily affect this parameter. For example, a worn or deformed GW can result in inconsistent material removal, leading to dimensional deviations. In addition, diameter, thickness and hole size of the GW must be in line with the desired tolerances [50]. If these parameters are off it will usually lead to inaccurate grinding results, specially regarding final dimensions and surface/subsurface integrity of the part [51].

Surface integrity a critical variable that reflects the texture or surface finish of the part after GP. Factors such as GW grain size, bond type, grinding speed and feed are some of the most important variables [52]. For example, larger grain size or a higher feed can lead to a rougher surface finish [53]. Likewise, an inappropriate grinding speed can result in an uneven surface texture and surface burns. Consistent surface roughness is crucial to meeting specific application requirements and ensuring the part performance [54].

Geometric shape deviation refers to the difference between the part shape after processing and the project. Factors such as GW wear/shape and chosen grinding method can influence this parameter [53]. For example, an excessively worn or irregularly shaped GW can lead to uneven material removal, resulting in a deviation from the intended geometric shape [55].

To ensure optimal grinding results, it is vital to regularly assess the GW condition. This assessment should include wear or deformation analysis and measurements of key parameters such as dimensions and roughness. Specialized equipment such as micrometers for dimensional accuracy, profilometers for surface roughness, and roundness testers for geometric shapes can be used for these measurements [56].

A comprehensive assessment of the abrasive tool condition is critical to maintaining high levels of performance. Regular inspections and measurements can help identify potential problems early, allowing for timely corrective action to ensure consistent, high-quality grinding results [57].

### Relation between grinding wheel condition and common grinding defects

Tool condition is a critical factor in the processing, impacting both most of the parameters related to the material removal. Various defects can arise due to poor GW condition, including longitudinal scratches, poor surface quality post-processing, inefficient material removal, uneven grinding, surface burns and changes of subsurface layer [58].

Longitudinal scratches are a typical defect that usually result from excessive GW wear or inadequate grinding speed, degrading the surface finish and resulting in a poorer surface quality. Regular GW dressing is the most effective tool to ensure uniform material removal and avoid this defect. Additionally, maintaining GW speed within an ideal range can help reduce scratch formation [59].

Poor surface quality after processing can occur due to several factors, such as unbalanced GW, inadequate grain size or insufficient cooling [60]. An unbalanced GW can lead to uneven material removal, resulting in a rough surface finish and even scratch marks. On the other hand, a GW with a grain size that is too large or too small for the application may result in decreased surface quality due to scratching or excessive heat generation. Insufficient cooling can also lead to excessive heat generation, causing thermal damage to the workpiece surface and subsurface [61].

Insufficient material removal is often due to binder vitrification or improper grinding parameters. Vitrification happens when the GW surface becomes smooth from excessive heat and pressure, affecting the binder properties. GW jamming occurs when chips clog the wheel's pores, reducing its efficiency [62].

Irregular grinding is a defect that can result from imbalance or uneven wear of the GW. This defect leads to inconsistent material removal across the entire surface of the part. Regularly balancing and dressing the GW are the most efficient ways to avoid this defect [63].

Surface burning is often caused by excessive heat generation during grinding. Factors such as high GW speed, inadequate feed rate or insufficient cooling can aggravate this problem. These burns not only degrade the part aesthetics, but also its mechanical and metallurgical properties, which can lead to premature failure [64].

Maintaining the GW in a proper condition is crucial to prevent these characteristic defects and obtain high-quality results. Regular inspections and proper maintenance practices, along with selection of the appropriate grinding apparatus for each application are essential to ensure consistent grinding performance [37].

## Sensor systems for monitoring tool condition in grinding

A controlled and predictable workpiece surface and subsurface is the main aspect of a process grindability [36]. Based on that, monitoring aspects such as GW clogging, abrasive friability, and workpiece temperature and roughness can significantly increase the GP's efficiency and sustainability.

**Fig. 3:**
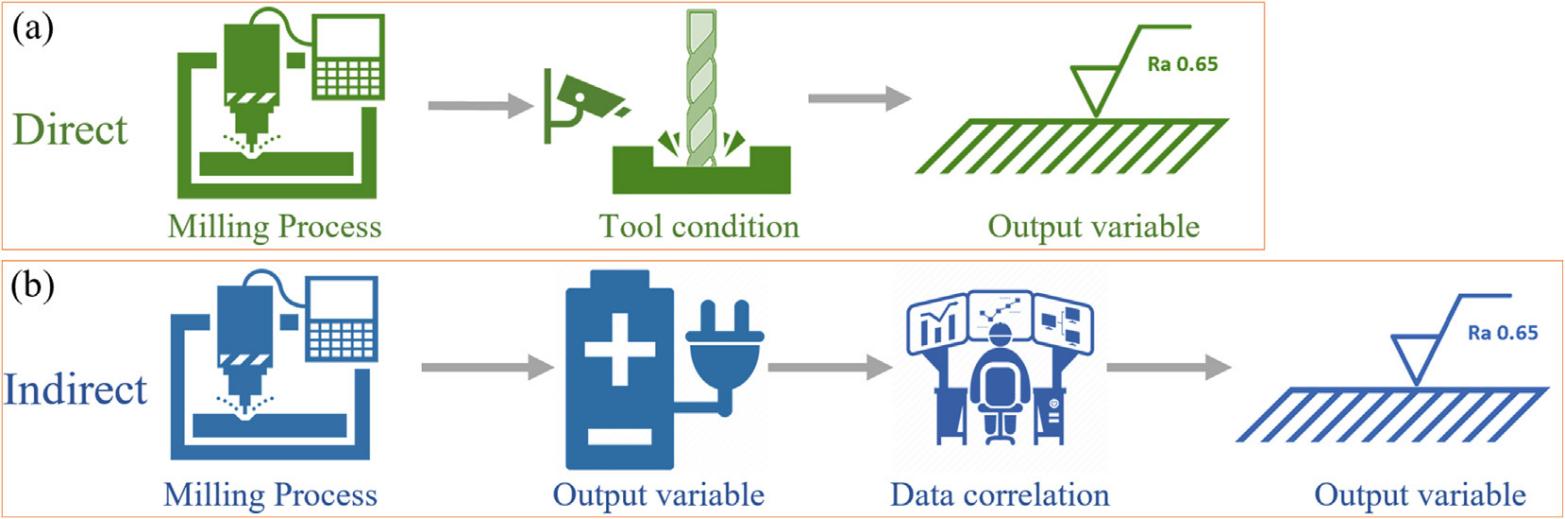
Representation of direct (a) and indirect (b) grinding monitoring.

The Fig. 3 highlights the differences between direct and indirect monitoring in the GP, focusing on how data is collected and analyzed to assess the condition of the grinding tool or process. As illustrated in Fig. 3, there are two primary approaches to monitoring the GP: direct and indirect measurements. While there is no strict boundary between these two methods, they can be distinguished based on how data is gathered and the variables being monitored [65].

In direct monitoring, data is collected directly from the grinding interface, typically by measuring variables closely linked to the final state of the workpiece or the condition of the GW. Sensors are placed at the tool/workpiece interface to measure parameters such as force, temperature, or AEs. These measurements provide real-time data about the GP, offering precise and immediate feedback on tool wear or process anomalies. This method is particularly advantageous in situations where immediate and accurate monitoring is critical, and access to the grinding zone is feasible [66].

In contrast, indirect monitoring involves gathering data from variables outside the grinding interface or when the process is already stopped. These variables are often correlated with output variables typically measured using direct monitoring. For example, sensors may measure vibration, current, or power consumption, which are influenced by the GP but do not require direct access to the grinding zone. Indirect monitoring is less intrusive and can be implemented in scenarios where direct access is impractical. While it may not provide the same level of precision as direct monitoring, it is effective in many practical applications where indirect indicators are sufficient [67].

In summary, direct monitoring focuses on gathering data directly from the grinding interface, while indirect monitoring relies on variables measured away from the interface or after the process has stopped. Both methods have their advantages, and the choice between them depends on the specific application, the accessibility of the grinding zone, and the level of precision required. This paper draws a clear distinction between the two methods to better evaluate their roles in monitoring the GP.

In the GP, the GW condition is usually a secondary concern than the workpiece surface and subsurface; thus, most monitoring efforts are directed towards the superficial and sub-superficial condition of the ground workpiece. The use of more traditional workpiece/tool monitoring methods such as dynamometry [68], infrared thermography (IRT) [69] and thermocouples (TCs) [70] still compose most of the research done. However, machine vision (MV) stands out as one of the most promising methods of direct monitoring both the workpiece [71] and grinding tool [72].

To avoid interfering in the actual GP, which can be laborious and compromise the system rigidity, other measuring methods present a growing demand in the industry. Among the most researched methods are vibration [73], AE [74], electric power [75], and electromechanical impedance (EMI) [76].

### Direct monitoring sensor systems for grinding tools

Directly measuring tool wear in grinding is particularly challenging compared to machining processes like milling and turning due to several factors: (i) higher grinding speeds, (ii) larger contact areas between the tool and workpiece, and (iii) smaller cutting zones due to abrasive grain size and multiple cutting regions that vary with the GW's characteristics. Additionally, removing the GW for optical or electronic microscopy analysis is impractical because of the difficulty and time required to reposition and balance it accurately. These challenges make traditional image acquisition methods unsuitable for industrial-scale monitoring.

**Fig. 4:**
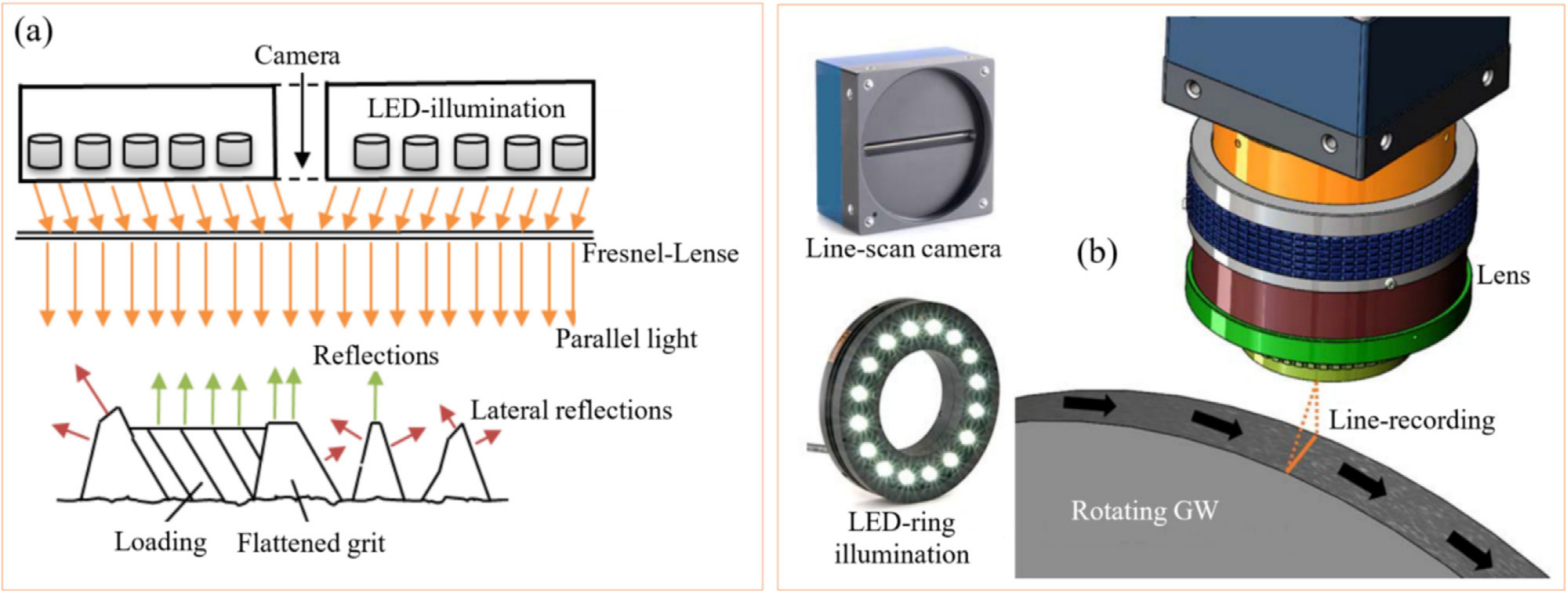
Image measurement principle for GW monitoring (a) and schematics of a line-recording image measurement setup (b).

Adapted from [80]

To overcome the aforementioned problem, the use of high acquisition rate cameras for monitoring the abrasive tool wear during the GP is pointed out as the most viable alternative for directly monitoring the degree of GW wear [77]. The increase in resolution and acquisition rate of both charged coupled device (CCD) camera [78] and complementary metal–oxide–semiconductor (CMOS) [79] resulted in an increased application of MV as a GW monitoring tool. An example of GW monitoring using MV is represented in Fig. 4.

Fan et al. [81] evaluated the use of a CCD camera to evaluate GW wear at an image capture rate of 30 Hz. The methodology applied by the authors for data processing was image binarization, followed by edge detection and wear evaluation. The authors reported that MV presented up to 1 μm of accuracy in assessing the GW wear compared to manual processing. Similar results were found by Feng et al. [82] using a similar methodology with CCD cameras capturing the GW surface images. According to Su et al. [72], a pivotal point to this method is to clean the GW before each image capture, as dust and chip could lead to lower accuracy in the image segmentation, as they could be mistaken as GW wear. The use of surface interferometry is another strategy to directly assess the tool condition during the GP. This technique consists of surface mapping, usually via laser or white light, providing a topographical representation of the tool surface, which based on different roughness parameters can be directly correlated with the tool wear. Yan et al. [83] evaluated the use of white light interferometry to characterize in three dimensions the condition of a GW using Fourier filtering to increase the accuracy of the measurements. The authors reported that the technique can be used to identify the abrasive size, angle, presence of adhered chip and dressing conditions. Similar results were reported by Lipinski et al. [84] used conventional laser interferometry, also inferring parameters such as grit size, spacing, and GW active area.

Pneumatic back-pressure measurements are other alternatives to measure the GW condition that, albeit less precise than laser and white light interferometry, present an overall higher cost-benefit for most heavy-duty applications [85]. This method, as described by Rahman et al. [85], consists in measuring the condition of the GW based on the back pressure using a pneumatic gauge of an air flow directed towards the grinding interface. Another methodology is proposed by Liu et al. [86] using the back-pressure measured by pneumatic probes coupled with pressure sensors, being similarly capable of assessing the GW wear as dressing state. Yin et. al [87] further improved the technique by coupling it with fuzzy neural networks (FNN) and Taguchi empirical analysis to create an online measurement method of GW wear with a confidence interval up to 98%. Those articles indicate that this technique is not only cost-effective, but also reliable for most situations were griding wheel monitoring is required.

Ultrasonic measurements are widely used in preventive and predictive general maintenance [88], being increasingly used in more specific applications such as machining. In general, for grinding applications the ultrasonic excitations are applied using piezoelectric (PZT) diaphragms transductors. Alexandre et al. [89] state that the GP is typically monitored using three transmission-reception configurations: Pseudo-Echo, Pitch-Catch, and Through-Transmission. Signals are analyzed using fast Fourier transform (FFT) to correlate specific frequencies with GW wear. One significant advantage, as noted by Alexandre et al. [89] is the ability to monitor GW wear and correlate signals with variables like workpiece roughness and burn.

### Indirect monitoring sensor systems for grinding tools

In grinding, tangential and normal forces result from interactions between abrasive grains and the workpiece at the grinding interface. These forces depend on grinding parameters, lubri-cooling conditions, and the average sharpness of the abrasives [65]. As most of the energy generated at the grinding interface flow towards the workpiece [30], machining forces are a clear indicator of how much heat will be delivered to the cutting interface, which usually is harmful to the surface and subsurface integrity [60]. Among the main challenges in measuring grinding forces is the dynamometer device's impact on overall stiffness. Couey et al. [90] evaluated the use of non-contact displacement sensors in an aerostatic spindle to monitor the cutting forces without affecting the system stiffness. The authors reported that the system could measure forces up to 25 mN while leading the system to only a 2 ηm spindle rotor deflection. In the machining process of delicate materials such as large and thin silicon wafers, monitoring the grinding forces is a crucial step regarding the machined surface and the overall part integrity. Zhu et al. [91] developed a 3D dynamometer based on PZT sensors to monitor the precision grinding of silicon wafers. The authors showed that the grinding forces directly correlate with the workpiece quality after the GP through experiments and finite element method simulations.

Unlike conventional machining, where heat is mainly dispersed in the chip, the smaller chip size and larger contact areas in grinding cause most of the heat to flow into the workpiece [37]. If not controlled, this heat can generate thermal damage such as grain growth and phase transformations, usually leading to surface and subsurface softening. Thus, monitoring cutting temperatures is an essential step to guarantee a proper process completion. In the literature, there are two main ways of monitoring grinding temperature: TCs and IRT cameras [92]. Wire TCs are usually fixed at the workpiece in a blind hole near the grinding interface using two methods: capacitance welding and cold-welding epoxy. García et al. [93] welded a k-type TC at a blind hole with 1 mm in diameter, at 3.5 mm from the workpiece edge, with 2.5 mm of depth. Using the data from the TC, the authors successfully modeled the heat flux at the workpiece using the Levenberg-Marquardt (LM) algorithm, finding the heat partition parameter through inverse methods. Similar results were found by Hadad et al. [94], which measured the energy partition of a GP under Minimum Quantity Lubrication (MQL) using epoxy to cold weld the TC in a blind hole at the workpiece.

Another way of measuring temperatures at the grinding interface is the use of IRT cameras. One of the main disadvantages in the use of thermal cameras to measure cutting temperatures is that its relatively low resolution makes it harder to measure temperature in regions immediately adjacent to the cutting interface, in addition to not being able to measure the temperature when cutting fluids accurately are present [92]. Brosse et al. [95] evaluated the use of IRT in investigating thermal fields under the ground surface using the Gauss-Newton inverse method. The authors reported that the use of IRT leads to a more accurate description of the temperature field than TCs. Like most body tissues, bones are highly susceptible to drastic temperature increases and irrecoverable damage. The use of IRT to monitor the GP of skull bones in neurosurgery was investigated by [96]. The authors found that the temperature increase was associated with the increase in rotational speed, feed rate, and cutting depth and that the use of IRT allowed a better tunning of the grinding parameters of the bones.

**Fig. 5:**
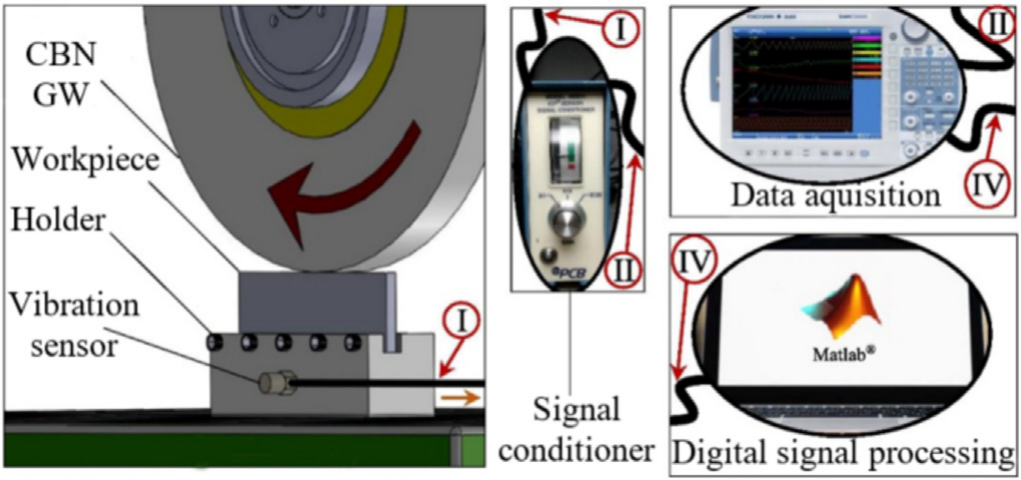
Vibration monitoring in grinding [99].

As in most machining processes, vibration is correlated with the system stiffness and usually results in poor surface finish and tool wear. In GPs, vibration control is especially critical since the main objective of the process is to provide a surface with tight tolerances regarding surface finish and dimensional form [97]. Vibration can usually be measured by PZT transductors [98], as those sensors provide the combination of stiffness and accuracy required for the high specific energy and low removal rate of the GP. Thomazella et al. [99] evaluated the use of PZT to measure self-vibration in the tangential GP of the AISI 1045 steel. The vibration sensor was magnetically positioned in the workpiece holder, as shown in Fig. 5. The authors reported that the use of PZT was effective in identifying chatter and its correlations with surface integrity.

AE is the system response to high-frequency (usually above 20 kHz) elastic oscillations [100]. The data gathering of AE is also usually performed using PZT transductors, and the processing of those oscillations is similar to those used in vibration analysis. However, as the oscillation frequency increases, a higher acquisition rate is needed and more computational time. Aulestia Viera et al. [74] evaluated the use of a low-cost PZT diaphragm transductor to monitor the AE signals (up to 300 kHz) in the GP of a ceramic workpiece (Al_2_O_3_) using a diamond GW. The performance of the PZT diaphragm transducer was compared with the DM-42 AE sensor from Sensis. The authors found over 80% coherence between the two sensors and a correlation between acoustic activity levels and surface roughness as grinding depth increased.

The increasing environmental awareness made many machining efforts move towards greener manufacturing [101]. One of the main aspects of increasing the sustainability of a process is to reduce energy consumption, especially in countries where the energy matrix is the fossil. Most methodologies use current and voltage sensors to measure the electric power spent during the processing [102]. As reported by Schischke et al. [103], by 2009, the GP represented around 28% of the energy consumption regarding CNC (computer numerical control) machines in EU-27.

**Fig. 6:**
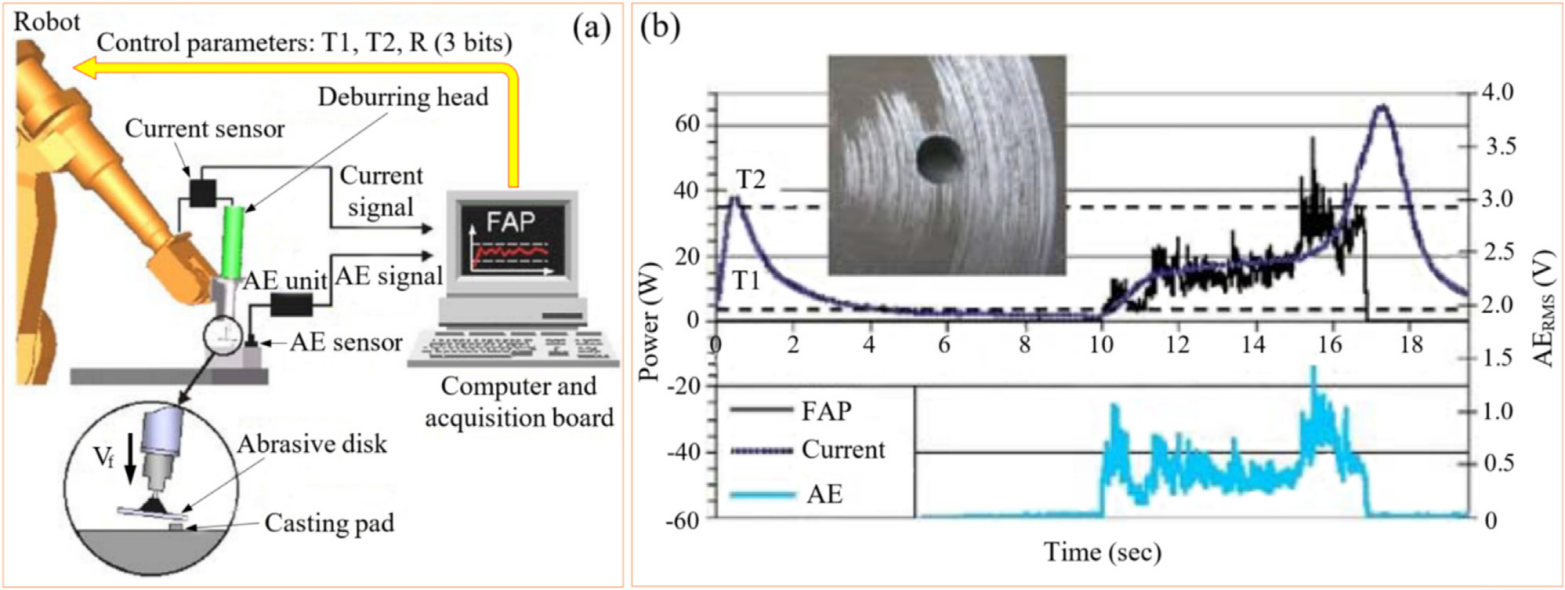
Experimental setup (a) and comparison between FAP, AE, and current signals (b) [104].

Oliveira et al. [104] compared robotic grinding monitoring using AE, electric current, and a new parameter called Fast Abrasive Power (FAP), as shown in Fig. 6a. Both AE and electric current exhibited similar trends in response to the GP (Fig. 6b). However, the FAP signal was more sensitive, detecting energy peaks when the disk first touched the workpiece and increased energy consumption after the robotic arm moved away, providing more comprehensive process monitoring. The FAP signal was more sensitive than AE and electric current signals because it combined the strengths of both signals while mitigating their individual limitations. The FAP signal is derived by modulating the electric power signal with the normalized AE RMS (Root Mean Square) dynamics. This fusion allows the FAP signal to inherit the fast response characteristics of AE and the reliability of the electric power signal. Overall, the FAP signal effectively combined the fast response of AE with the robustness of power signals, making it more sensitive and reliable for detecting and responding to process variations in grinding operations.

**Fig. 7:**
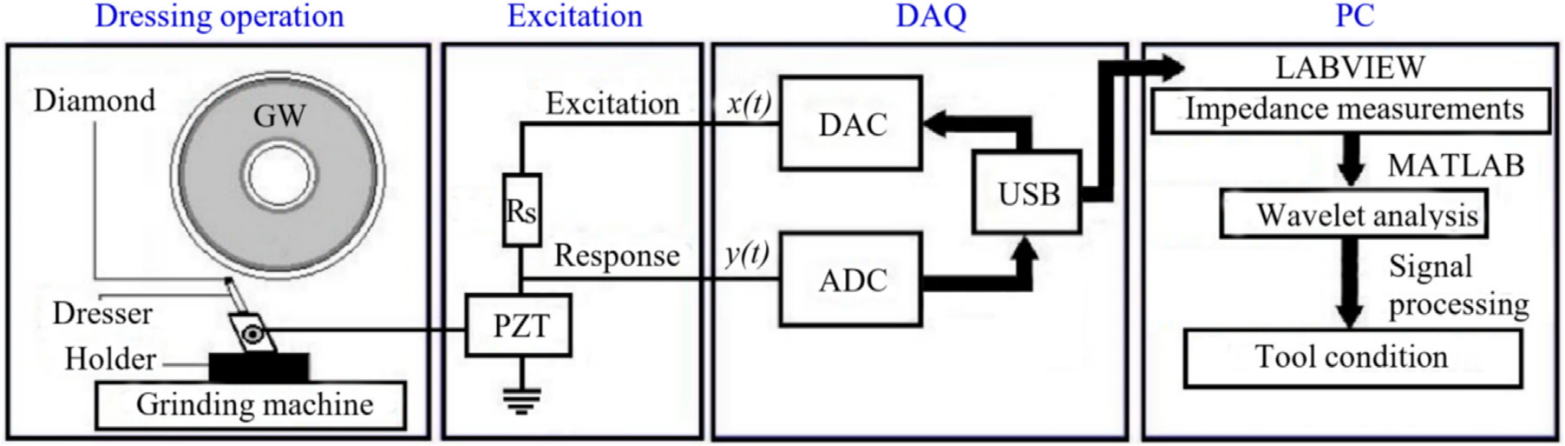
Dressing process monitoring using EMI and wavelet analysis [107].

EMI is a well-established non-destructive monitoring technique mainly used in structural health applications [105]. Recently this technique is being applied to monitor machining processes, especially grinding, due to its lower MRR and higher susceptibility to thermal-induced microstructural changes [106]. Another application of EMI in grinding is to monitor dresser and abrasive tool conditions during the GP. As Junior et al. [107] studied, EMI can be monitored using low-cost PZT transductors, and wavelet transforms, as illustrated in Fig. 7. The authors reported that EMI signals could be successfully applied to estimate GW condition and dresser wear, helping to select the appropriate dressing conditions for the operation.

### Ai-driven sensors for grinding tool monitoring

A large amount of data from the many possible ways of monitoring the GP can sometimes be challenging to process and get helpful information. AI and other advanced statistical techniques are the main alternatives to help in this task [108]. Some of the most commonly evaluated techniques include Fuzzy logic [109], adaptative neuro-fuzzy systems (ANFIS) [110], regression and decision trees [111], k-nearest neighbors (kNN) [112], artificial neural network (ANN) [113], support vector machine (SVM) [114], and genetic algorithms (GA) [115].

In addition to the commonly evaluated techniques, AI-driven sensors also incorporate advanced signal processing methods to enhance the monitoring and analysis of the GP. For example, convolutional neural networks (CNNs) can be employed to automatically extract meaningful features from complex signals, such as AE or power signals, without requiring extensive manual preprocessing [116]. These signal processing techniques enable more accurate detection of process variations, improve the interpretation of sensor data, and facilitate real-time decision-making. By combining AI algorithms with sophisticated signal processing, monitoring systems become more robust, efficient, and capable of handling the complexity of modern grinding operations [117].

Mahata et al. [118] monitored the GW sharpness using electrical power and vibration from triaxial accelerometer data. After the data gathering, it was processed using the Hilbert Huang transform (HHT) and SVM to perform time–frequency adaptative analysis of both evaluated signals. After the initial processing, the signal-specific features were identified using a Random Forest (RF) algorithm. The authors reported that the proposed method allowed to distinguish between sharp and worn GWs and to evaluate the intermediate states that exist during the tool wear process.

Moia et al. [113] investigated the monitoring of an Al_2_O_3_ GW during dressing using AE. They employed a neural network with the LM algorithm and RMS values of AE signals as inputs. The study found that this method could accurately evaluate the GW's state during dressing, achieving nearly 100% accuracy.

**Fig. 8:**
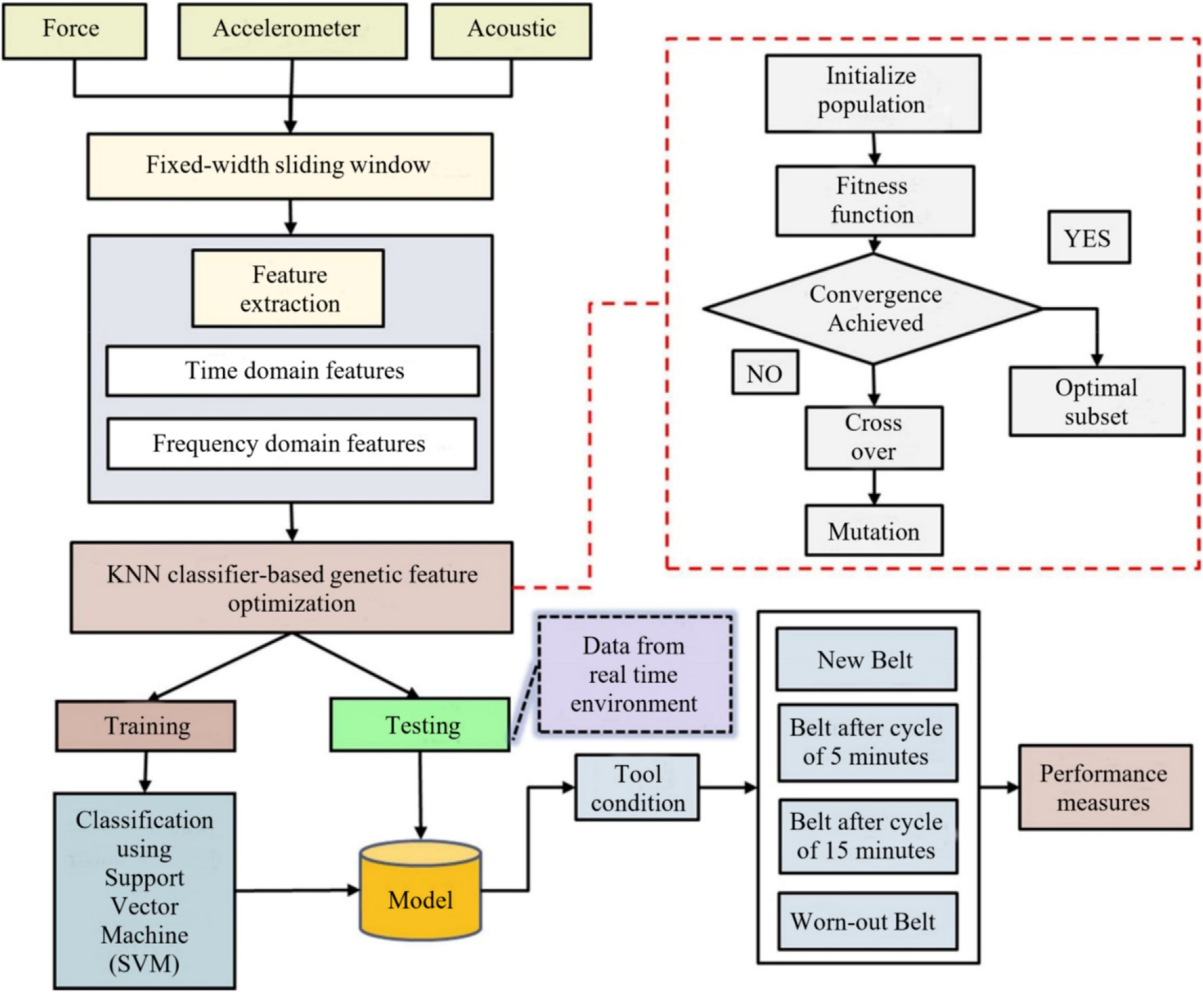
Methodology for tool wear monitoring in belt grinding using SVM and GA to analyze grinding force and AE data [15].

**Fig. 9:**
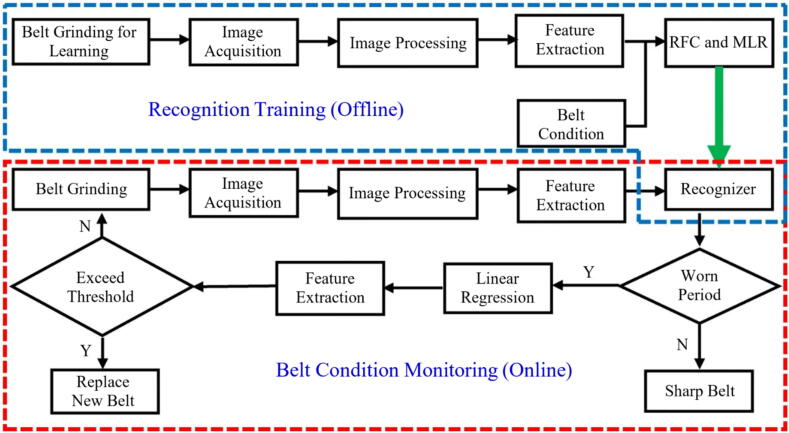
Methodology for wear monitoring using MLR and RF classifier to process MV data [111].

Pandiyan et al. [15] evaluated the use of SVM and a kNN classified genetic algorithm to monitor and predict tool life in the belt GP, using AE, vibration, and grinding force signals as data input. The methodology used to predict tool wear is illustrated in Fig. 7, using kNN. The authors reported that using kNN as a classifying tool for implementing the genetic algorithm reduced the number of features from a pure GE analysis from 162 to 26 features. The proposed methodology (Fig. 8) predicted the tool wear in the evaluated process with an accuracy of up to 94.7%. Similarly, Oo et al. [111] evaluated the tool wear in the belt GP using MV, combining a model with multiple linear regression (MLR) and a RF classifier to detect the different wear states. The flowchart illustrated in Fig. 9 represents the steps used by the author in the monitoring process. The authors found that correlating belt wear with abrasive grain area features resulted in a detection accuracy of over 96% using their chosen methodology.

## Indirect tool condition monitoring in grinding

### In-Cycle measuring sensors and applications

The conditioning of the GW affects both the finish and the surface and sub-surface integrity of the machined component. Therefore, monitoring is crucial to determine the optimal time for dressing to maintain the abrasive tool's cutting efficiency. Various methods exist for monitoring GW wear, including “between cycles” and “in-cycle” techniques. “Between cycles” refer to measurements taken after machining, that is, in the case of monitoring the conditioning of the GW, the main methods are roughness analysis [86], photos of the abrasive GW [119], [120] and wear measurement [121], [122]. The “in-cycle” ones refer to the monitoring performed during machining, some of the main ones being through power and electrical current [123], [124], force [125], vibration [126] and AE [109].

### Conventional sensor systems overview

Various sensors could be used to estimate the condition of the abrasive GW, with their selection depending on factors such as cost and ease of installation [72]. In the case of sensors used for in-cycle measurements, the main ones are the dynamometer (force), accelerometer (vibration), AE sensors and power sensors. In Table 1 are shown the advantages and limitations of each of these sensors. More information about these and their functionality is presented in the next sections of this article.

**Table 1:** Advantages and limitations of the main sensors used for monitoring GW conditioning in cycles.

**Sensor**	**Main advantages**	**Main limitations**
Force	− It is one of the most used because the increase in GW wear directly influences the increase in cutting efforts. Furthermore, it is a low-cost method [127].	− Because of the inert masses in the system, force signals typically exhibit a low-pass filter characteristic, making them insensitive to minor process variations. [8].
Power	− Obtaining power values is easy, in addition to being possible to obtain force values through power [128]; − It is easy to install the system and has a low cost [37].	− Studies have shown that the dynamic response of a power sensor coupled to the machine axis has limitations [37].
Vibration	− They are low cost, easy to use and highly reliable [129], [130]. − Vibration sensors have high sensitivity [131].	− The signal amplitude varies depending on the sensor's position, and if the sensor is placed near the work zone, the acquired signal will exhibit significant variability [129].
AE	− The AE signal has a frequency range that exceeds that of machine tool vibrations and environmental noise[132]. − It has high sensitivity [133]; − Low cost and easy to employ [37].	− Small changes in the system, such as sensor positioning and cutting fluid flow, can interfere with the signal [134].
Temperature	− Provides direct measurement of heat generated during the GP, which is critical for monitoring thermal damage [135]. − Non-invasive and easy to implement [136].	− Limited to monitoring surface temperature and may not provide detailed insight into subsurface thermal effects [137]. − Sensor placement and cooling fluids can affect accuracy [138].
Ultrasonic	− Capable of detecting microstructural changes and cracks in the workpiece [89]. − High resolution and sensitivity [139].	− Requires coupling medium (e.g., gel or liquid) for effective signal transmission [89]. − Signal interpretation can be complex and requires expertise [139].
Optical	− Non-contact measurement method, minimizing interference with the process [140]. − Can provide high-resolution surface measurements [141].	− Sensitive to environmental factors such as lighting conditions, dirt, and debris [142]. − Typically higher cost compared to other sensors [143].
Laser	− Extremely precise and capable of measuring surface roughness, dimensional changes, and tool wear [144]. − Non-contact and fast measurement technique [144].	− High initial cost and maintenance requirements [144]. − Performance can be affected by surface reflectivity and environmental conditions such as dust or vibrations [144].

#### Applications in conventional and AI-driven Contexts

**Fig. 10:**
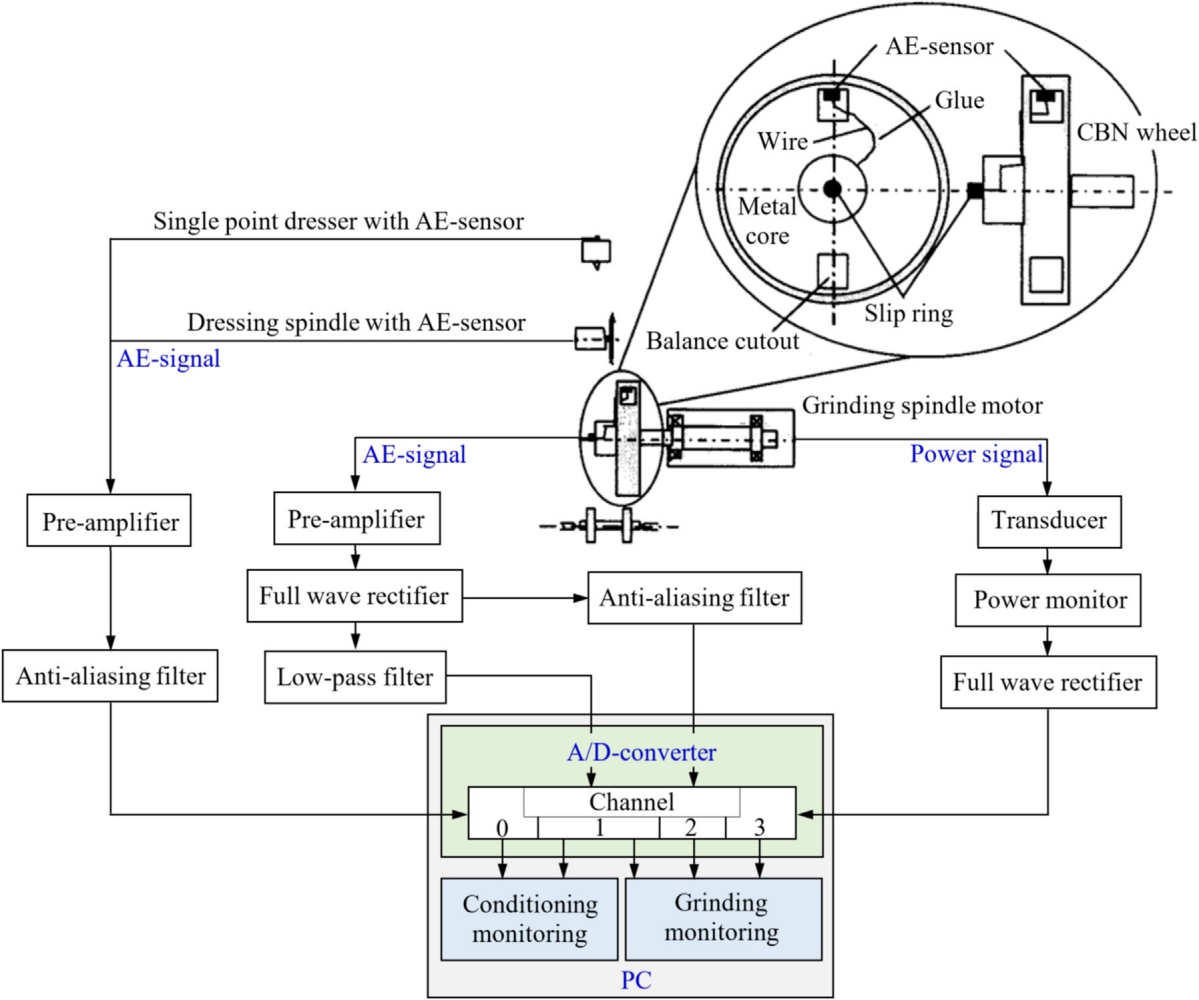
System used to monitor abrasive GW wear through power and AE [123].

Several researchers have carried out research studying the various sensors capable of monitoring GW conditioning in order to understand the advantages and disadvantages of each one, in addition to identifying under which conditions one or the other is more or less efficient in performing this functionality. From the analysis of different researches it is possible to see that some studies have indicated efficiency in combining different sensors. In external grinding tests, Karpuschewski et al. [123] combined power signals with AE to analyze the conditioning of the GW (Fig. 10). The power values were acquired through a transducer coupled to the motor spindle while the AE signals through an AE-sensor and employing an analog-to-digital (A/D) converter. Furthermore, the analysis took place using AI methods. The authors highlight that integrating data from various sensors enables the monitoring of the GW's condition, as signal disturbances occur when the GW reaches the end of its lifespan.

**Fig. 11:**
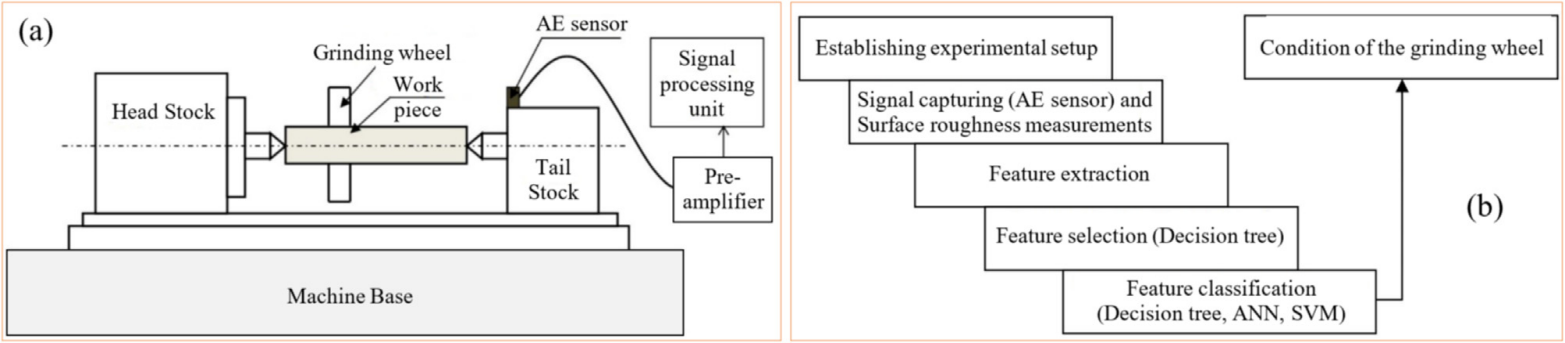
System for carrying out experimental tests (a) and methodology for analyzing the results obtained (b) [146].

The results found in research carried out by Subrahmanya and Shin [124] also indicated good effectiveness of the power signals (obtained through a Hall effect-HE sensor) in monitoring the conditioning of the abrasive GW, that is, through the power values it was possible to identify the magnitude of GW wear, the values obtained being close to the experimental values measured. During research developed by Chen and Limchimchol [114], the authors observed efficiency in using the results of force in the GP to predict the useful life of the GW. In other words, this study demonstrated that there is a relationship between the cutting efforts developed in the process and the GW wear rate. In addition, the SVM methodology was used, which proved to be efficient in classifying the different wear conditions of the abrasive tool. Tian et al. [145] used the power signals to understand the conditioning of the abrasive wheel during surface grinding test of the nickel alloy 718. The portable power system used includes a PPC-3 power meter, a data acquisition system (cDAQ 9171 and NI 9125), and a computer with analytical software. The authors discovered a correlation between power signals and GW conditioning, allowing for the prediction of when dressing is needed. High power values were linked to the loss of the abrasive wheel's cutting capacity, indicating the need for dressing. Arun et al. [146] performed grinding tests and used AE signals (obtained by means of a PZT sensor) to monitor the evolution of GW wear. Additionally, ML techniques, such as decision trees, ANN, and SVM, were employed to predict the condition of the GW. The results obtained indicated the efficiency of monitoring GW conditioning with AE, and the use of the SVM showed better accuracy in predicting the wear of the abrasive tool compared to decision tree and ANN. In Fig. 11 are shown the system used to carry out the experimental tests and the methodology for analyzing the results obtained.

**Fig. 12:**
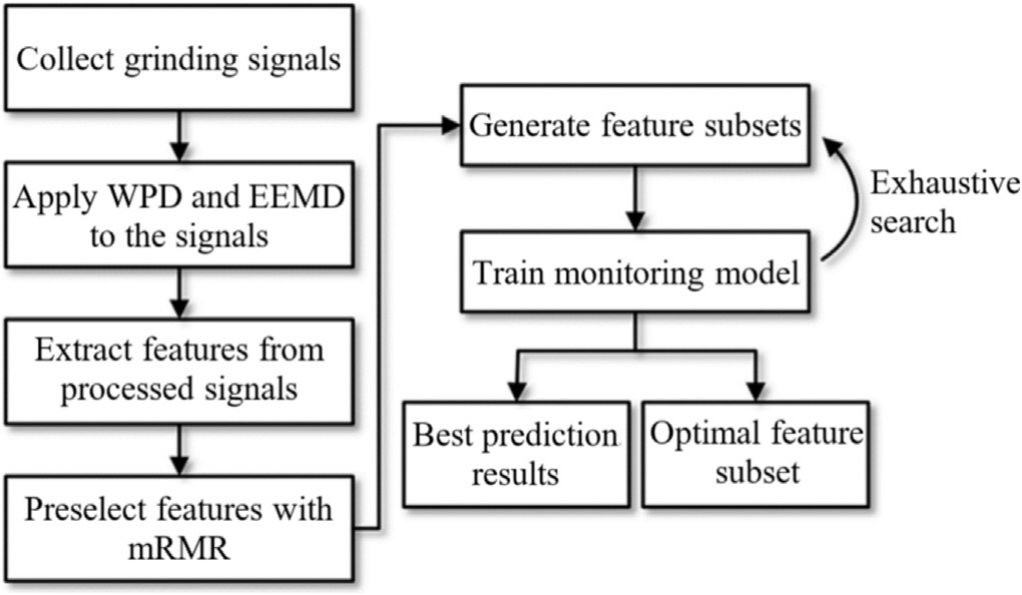
GW conditioning monitoring flowchart [147].

To evaluate abrasive GW wear during the GP, Guo et al. [147] analyzed signals from various sensors, including a dynamometer, accelerometer, and AE sensor. They used two models to predict GW conditioning: the Long Short-Term Memory (LSTM) model and the RF model. The results indicated that the RF model was more accurate in predicting GW wear. Fig. 12 ilustrates the flowchart for GW conditioning monitoring, using tools like Wavelet Packet Decomposition (WPD), Ensemble Empirical Mode Decomposition (EEMD) and Minimum Redundancy Maximum Relevance (mRMR).

Zhang et al. [148] developed a GW wear monitoring system using an interval type-2 fuzzy basis function network (FBFN). They compared experimental results obtained from a power sensor, accelerometer, and AE sensor. The model effectively determined the GW's condition and the optimal time for dressing. In another study by Lopes et al. [149], AE was used to monitor the surface integrity of the abrasive wheel during dressing tests. By analyzing signals in the time–frequency domain with a short-time Fourier transform (STFT), they were able to understand changes in the topography and cutting capacity of the GW's edges.

#### Summary of the Subsection

There are several methods for monitoring abrasive GW conditioning, some “between cycles” and others “in-cycle”. Usually the “in-cycle” ones are preferred because they are monitored while the machining takes place, that is, they are able to detect any “disturbance” in the process and stop it before it compromises the component being machined. The selection of the best methodology will depend on several factors, from cost to ease of installation of the sensors. Thus, whatever type of output variable will be evaluated to monitor GW wear must be chosen considering the advantages and disadvantages of each. In addition, it is noteworthy that AI has contributed a lot to the development of this area as it often makes measurements cheaper and more accurate. In Table 2 are shown the advantages and limitations of each of these sensors.

**Table 2:** Methods of monitoring the GP using conventional sensor systems.

**Grinding Process**	**Year**	**Main application**	**Sensors**	**Reference**
External grinding	2000	GW conditioning analysis	Power sensor, AE sensor	[123]
Plunge grinding	2008	Automated sensor selection and monitoring	HE sensor	[124]
Surface grinding	2017	GW conditioning during nickel alloy 718 grinding	Power sensor	[145]
Cylindrical grinding	2018	Monitoring GW wear	AE sensor	[146]
General grinding	2019	GW wear prediction using AI	Dynamometer, accelerometer, AE sensor	[147]
General grinding	2020	GW wear monitoring and optimal dressing prediction	Power sensor, accelerometer, AE sensor	[148]
Dressing tests	2021	Surface integrity of abrasive wheels	AE sensor	[149]

### Force sensors

In the GP, the cutting edges of the abrasive particles that compose the GW wear down. As a result, the tool's cutting capacity decreases, leading to an increase in the cutting effort required for machining [58]. In this sense, monitoring the cutting forces can be used to monitor the conditioning of the GW [118]. Nakai et al. [128] highlights that indirect monitoring is normally used because it is not necessary to interrupt the machining to measure this output parameter.

#### System overview

Although in some studies the forces developed in the process are normally obtained using PZT sensors, these sensors have a high cost and, therefore, their employability in industrial sectors is sometimes unfeasible. An alternative that has been used is the use of plate or ring force sensors, which are composed of quartz components [150]. Despite this, among the force measurement sensors, the dynamometer has been widely used in several machining research, such as in the GP [151], [152], turning [153], [154], milling [155], [156], among others. Daniyan et al. [157] highlight that because the dynamometer is a PZT sensor, it has high sensitivity and works at high frequencies, being able to detect changes easily and quickly in cutting efforts. In this way, it is possible to use this information for several purposes, including the monitoring of tool conditioning. As stated by Hanif et al. [158], the operation of a dynamometer consists in capturing the elastic deformation rate of the flexible mechanical component, thus resulting in obtaining the cutting efforts of the process. The authors also point out that most dynamometers are produced using a strain gauge or PZT material.

#### Applications in conventional and AI-driven Contexts

Lezanski [98] evaluated the wear of abrasive GW in the grinding of AISI 1045 hardened carbon steel. For the prediction of GW conditioning, a system based on ANN and fuzzy logic combining the signals from different sensors was employed. According to the authors, the use of a neurofuzzy algorithm is one of the only alternatives in cases where there are several input variables, such as for the GW condition. It is noteworthy that the use of this neurofuzzy model provided an accuracy of 83.3%.

Kwak and Ha [159] used the force signals of grinding tests to monitoring the conditioning of the GW and, consequently, define the best time to perform the dressing of the abrasive tool. The results obtained indicated that the cutting efforts linearly increased with the increase of machined workpieces, with force values beginning at close to 45 pieces to vary drastically indicating the time of dressing. Furthermore, the authors emphasize that for a more precise definition of the dressing time it is necessary to use a discrete wavelet transform used Daubechies wavelets. Saleh et al. [160] and Kannan and Arunachalam [161] also used a similar methodology, that is, the analysis of force signals to define the optimal dressing moment, and the results indicated this method efficiency.

Feng et al. [127] developed a study related to GW wear in micro-GP and acquired several signals for this purpose, among them the obtaining of force signals through a Kistler 9256C1 dynamometer. The authors emphasize that in the case of microgrinding, tool wear does not cause great variations in the measured signals, indicating great difficulty in monitoring GW conditioning. However, the combination of force and vibration signals proved to be efficient for this purpose.

**Fig. 13:**
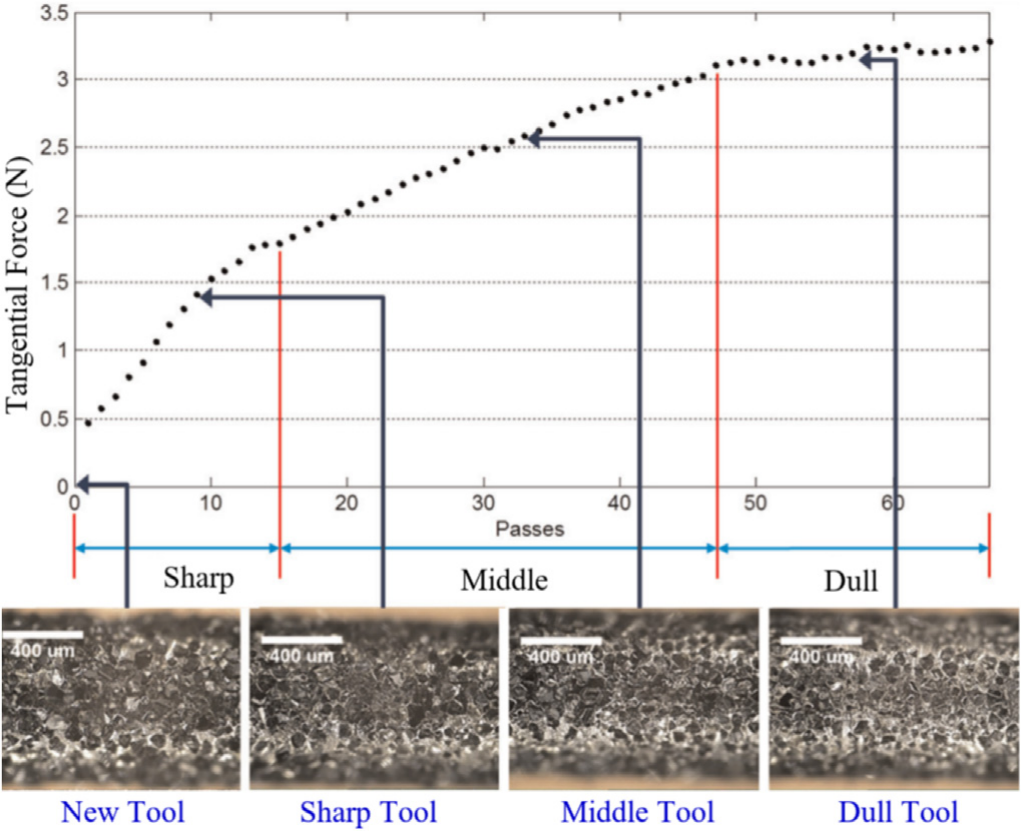
Average tangential grinding forces measured and corresponding photos of the grinding tool [125].

**Fig. 14:**
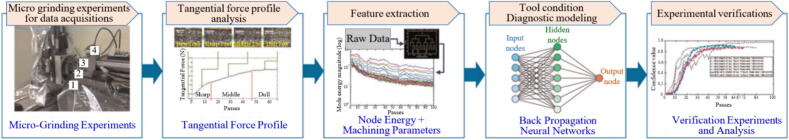
Schematic overview of the modeling and validation process for diagnosing micro-grinding tool conditions. .

Adapted from [125]

In a study on GW wear in micro-GPs, Lee et al. [125] used load cells to measure force signals. Tangential forces were analyzed using WPD, and numerical confidence values were calculated using a backpropagation neural network (BPNN). The results are shown in Fig. 13, demonstrate that as the abrasive wheel wears, the cutting efforts increase. In Fig. 14, an overview of the modeling and validation of the diagnosis of the abrasive GW conditioning proposed in this study is presented. Based on experimental tests for validation, it was possible to verify that the proposed model is efficient to be used in several cases in GPs, while some other models that have been studied are restricted to just a few cutting conditions.

**Fig. 15:**
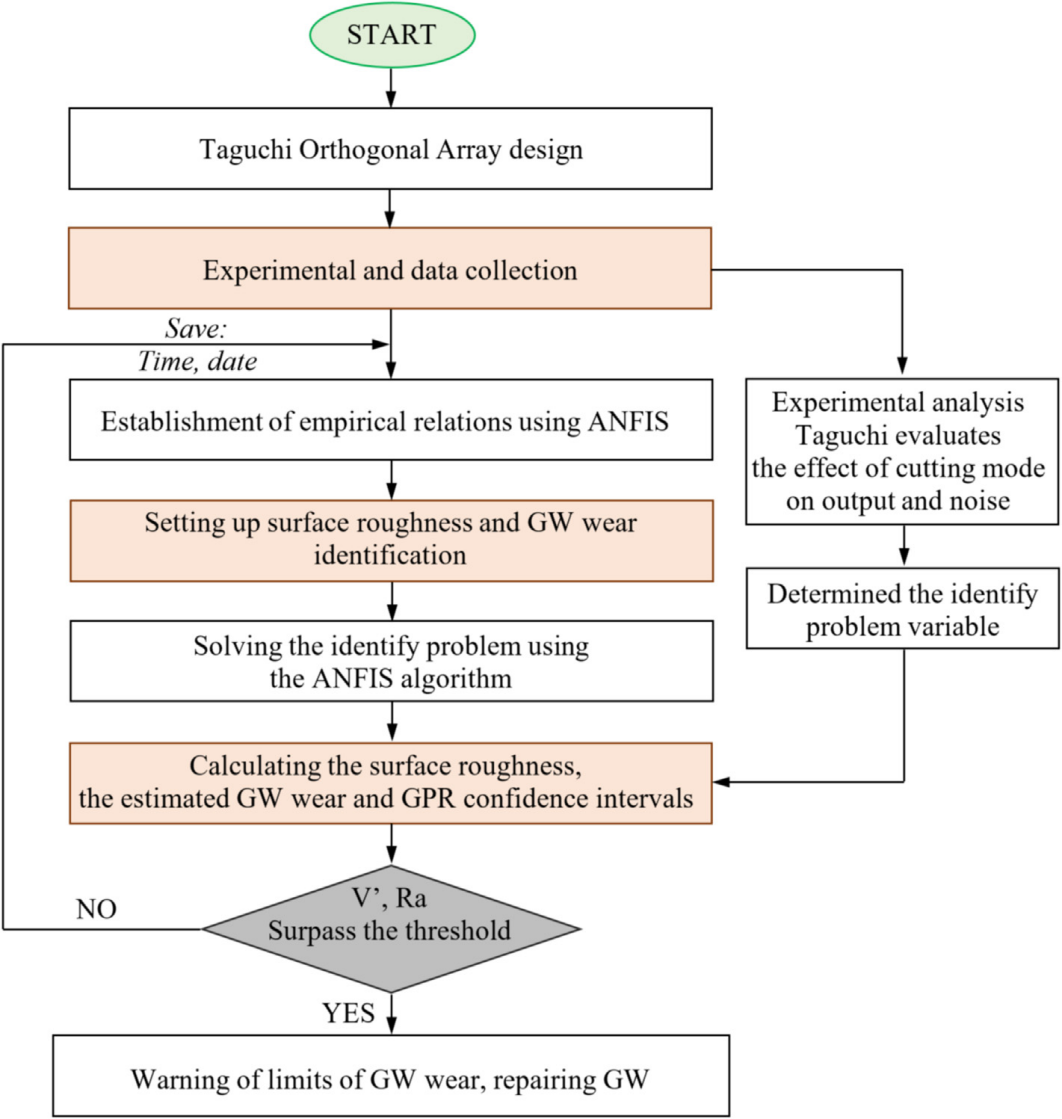
A new effective operator for the hybrid algorithm to solve online monitoring. .

Adapted from [162]

In their grinding tests on the Ti-6Al-4 V alloy, Nguyen et al. [162] investigated GW wear by analyzing grinding force signals. They employed an adaptive neural fuzzy inference system (ANFIS), Gaussian process regression (GPR), and Taguchi analysis for their study. In Fig. 15 the operator for the hybrid algorithm to solve online monitoring is shown. From the comparison with the experimental results obtained, it was possible to verify that the proposed model is efficient in determining the conditioning of the abrasive GW. In addition, this wear-prediction capability has an extremely low average error (0.31%) and 98% reliability.

#### Summary of the Subsection

The analysis of grinding forces stands out in monitoring abrasive GW conditioning since GW wear indicates an increase in cutting efforts and through the study of force signals it is possible to understand GW conditioning [125]. However, it is observed that the sensors normally used, such as the dynamometer, have a high cost, making its use unfeasible in different situations [150]. A promising line of research is the use of AI to predict GW conditioning. Several papers have indicated that the use of AI has shown accurate results regarding the monitoring of abrasive GW wear [162]. In Table 3 are shown the advantages and limitations of each of these sensors.

**Table 3:** Methods of monitoring the GP using force sensor systems.

**Grinding Process**	**Year**	**Main application**	**Sensors**	**Reference**
Grinding	2001	GW wear monitoring using ANN and fuzzy logic	Multiple sensors (neurofuzzy)	[98]
Micro-grinding	2009	Tool wear monitoring for ceramic materials	Kistler 9256C1 dynamometer	[127]
Micro-grinding	2015	GW wear monitoring using force signals and BPNN	Load cells	[125]
Grinding	2015	GW wear monitoring using cutting forces	Dynamometer	[151]
Grinding	2015	GW wear monitoring using cutting forces	Dynamometer	[153]
Grinding	2010	Optimal dressing prediction using force signals	Dynamometer	[160]
Grinding	2018	Optimal dressing prediction using force signals	Dynamometer	[161]
Grinding	2021	Ti-6Al-4 V alloy GW wear prediction with AI	Force sensors, ANFIS, GPR	[162]

### Current and power sensors

Monitoring the GP with current and power sensors is widely utilized due to its low cost and ease of installation. It requires no additional fixing devices or tool modifications, making it very popular in the industry. Moreover, it helps identify and subsequently reduce energy consumption.

Grinding can be likened to milling, but with numerous abrasive grains instead of cutting teeth [163]. The thickness of the chips removed by abrasives varies from zero to the maximum chip value, similar to a milling cutter [164]. Each abrasive grain removes chips of only a few microns in size [165], but since many abrasives work simultaneously, the total chip volume is significantly larger [166]. Abrasive wear and detachment from the grinding stone are critical factors affecting the GP [14]. Research on the machine, cutting tool, and material has underscored the importance of analyzing and accurately measuring current and power consumption during machining [167], [168], [169]. Despite extensive studies, some issues with the grinding material during the process remain unresolved.

#### System overview

Pimenov et al. [170] describe the primary method for measuring current and power as determining the current drawn by the machine tool's main shaft and calculating power based on the relationship between power, current, and resistance. Power consumption can be derived from cutting forces using the equation: power equals velocity times force. This is measured with an electrical power sensor in the spindle motor. Installing power monitoring equipment is straightforward, requiring the coupling of current transducers and voltage sensors. The power signal directly correlates with the forces involved, with changes in load reflecting changes in power. The analysis involves interpreting signal patterns from various operating conditions and abrasive tools. Notably, power is a more sensitive variable than current, particularly under low load conditions, making it more commonly used.

**Fig. 16:**
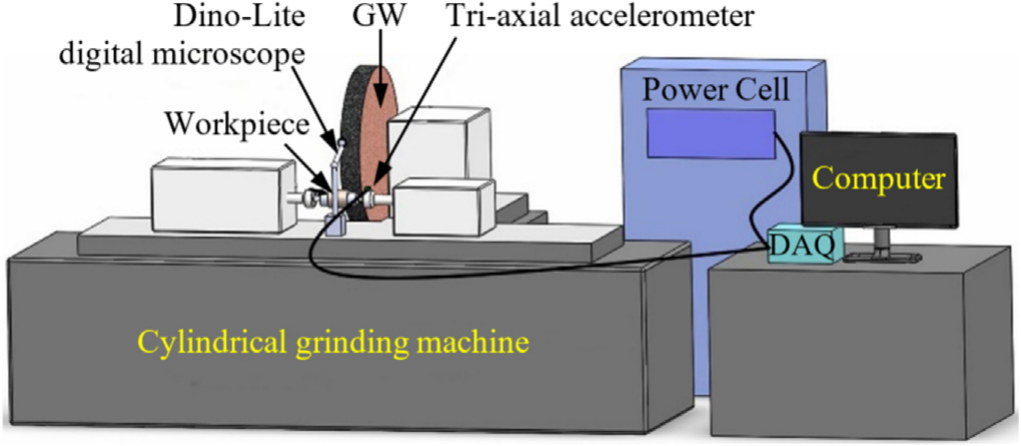
Example of experimental setup for power monitoring in grinding [118].

In the specific case of monitoring diamond rollers under low loads, Tingbin et al. [171] demonstrated that when the electric spindle's power is too low, the collected current signal value does not change significantly, making it insufficient to assess the grinding state. Fig. 16 displays an example of an experimental setup for power monitoring in grinding.

**Fig. 17:**
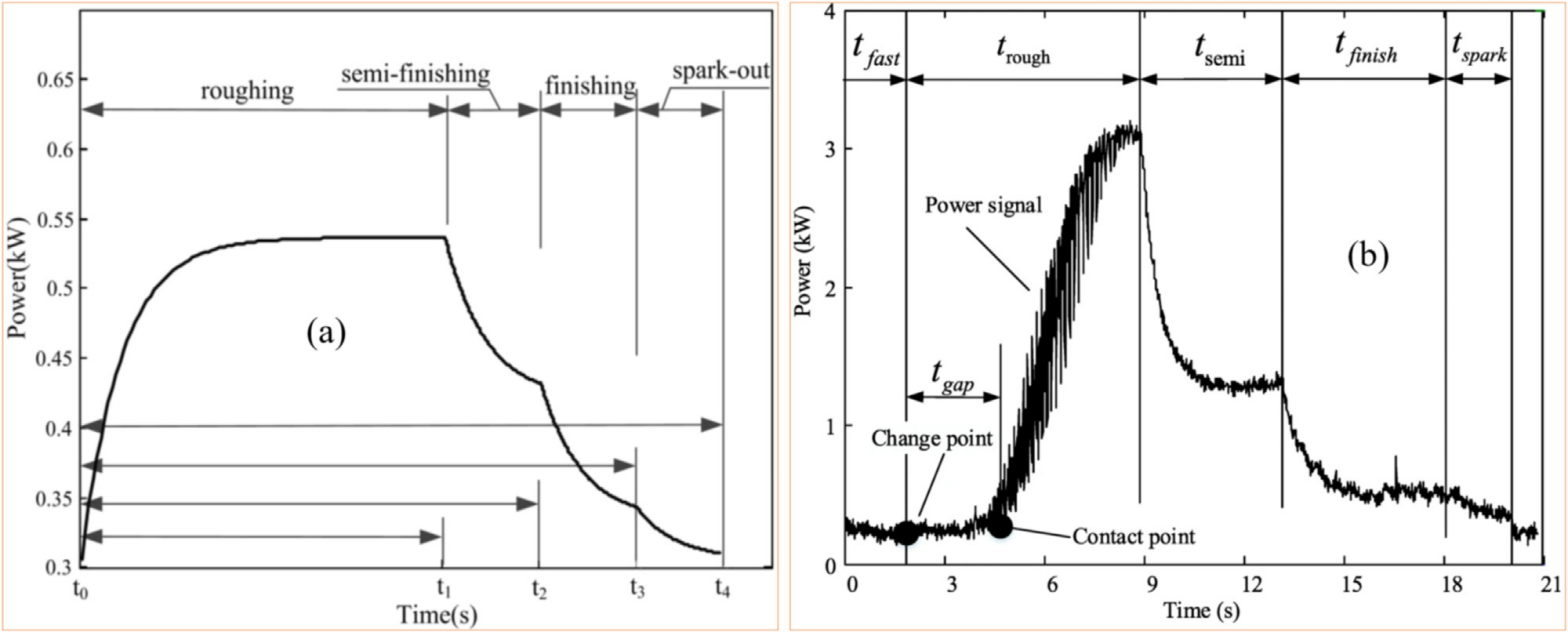
Grinding stages as a function of power signal [173].

Typically, the power signal contains noise. To eliminate this, it is necessary to apply a low-pass filter after identifying the frequency range using the FFT [172]. The different stages of grinding can be identified in the power signal. In Fig. 17a, the ideal pattern of the curve is shown, covering roughing, semi-finishing, finishing, and spark-out, while Fig. 17b displays the real signal.

**Fig. 18:**
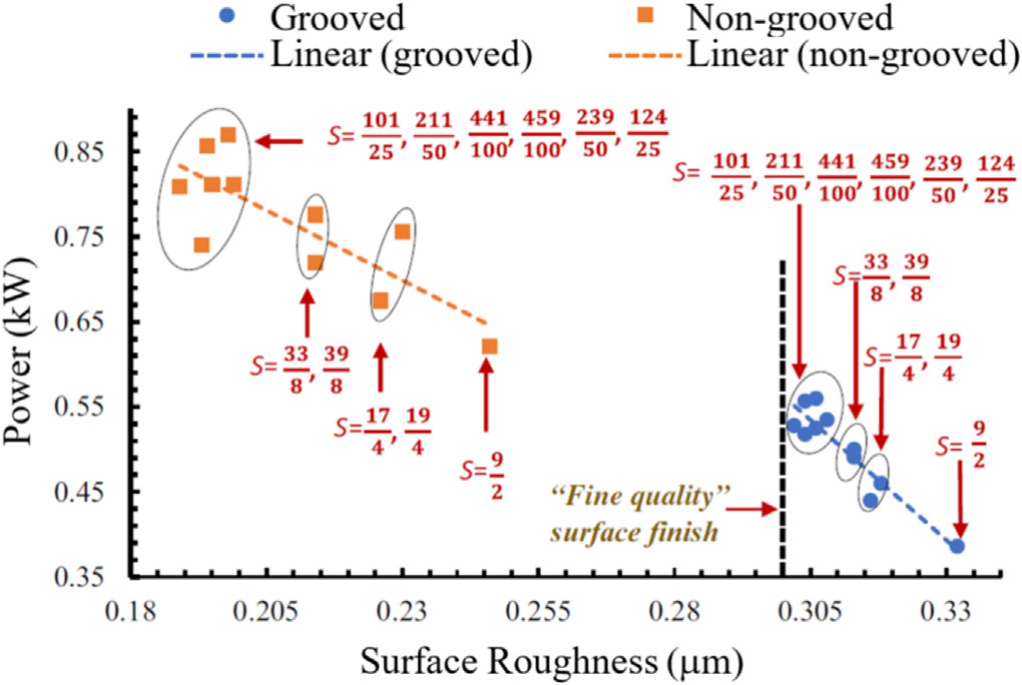
Correlation between power and surface roughness [174].

**Fig. 19:**
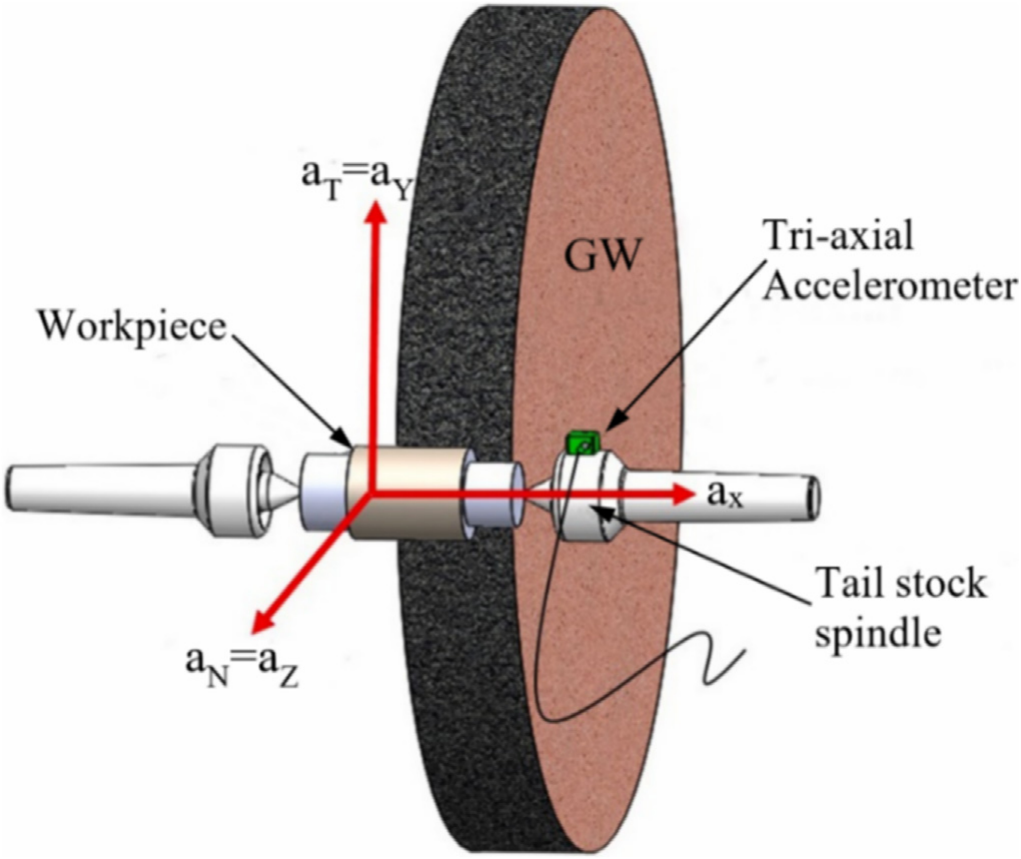
Placement of the tri-axial accelerometer [118].

**Fig. 20:**
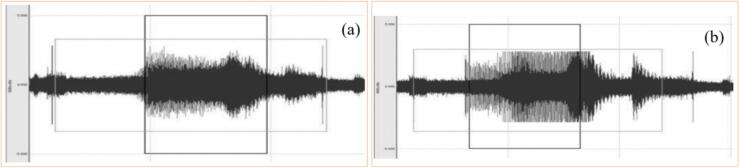
Vibration level recorded in the GP after GW dressing (a) and with worn GW (b) [180].

#### Applications in conventional and AI-driven Contexts

Recent studies utilizing current or power monitoring to examine the GP focus on correlating these variables with final desired parameters. For example, Garcia et al. [174] assessed the relationship between power and surface roughness during grinding, as shown in Fig. 18. Wang et al. [172] proposed a new exponential polynomial model of surface roughness as a function of the power signal. They emphasize that grinding variables and GW conditions can be indirectly associated with the power signal. In other words, the variation in the power signal is capable of describing the dynamics of the manufacturing process. This enabled the development of an online diagnostic method. Onishi et al. [175] were able to calculate the removed volume of the workpiece from the power consumption of the wheel motor in the cylindrical GP.

**Fig. 21:**
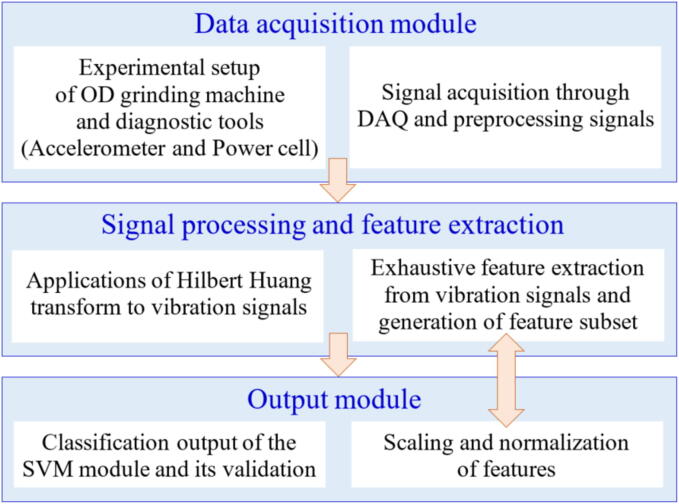
Flowchart of the methodology [118].

Shi and Attia [176] state that power magnitude indicates material grindability and various grinding phenomena, such as wheel dulling, collapsing, and grinding fluid burnout in creep-feed grinding. Total power includes net power, correlated with net forces, and non-grinding power, which arises from fluid drag and spindle bearing friction. Since non-grinding power is unrelated to grinding force, their work introduced a new method to measure non-grinding components.

Wang et al. [172] presented an innovative method for online prediction of GW condition and surface roughness during the precision grinding of fused silica ceramic matrix composites (SiO_2_f/SiO_2_). By leveraging power signal monitoring, a warm-cold density chart of specific grinding energy (SGE) is introduced to visualize and predict GW wear states. An exponential polynomial model is proposed, incorporating grinding parameters and cutting power to predict surface roughness more accurately, particularly in worn-out GW conditions. Experimental results across five grinding scenarios demonstrated the effectiveness of the proposed method, with significant improvements in prediction accuracy, as reflected by reduced residual errors (REs), lower mean square error (MSE), and a higher Pearson correlation coefficient (r = 0.8710). This approach provided a cost-effective and non-intrusive solution for real-time monitoring and optimization of GPs, though further research is recommended for complex geometries and additional quality indicators.

It is also possible to combine the power signal with the vibration signal to predict the process behavior, as demonstrated by Mahata et al. [118]. Using a HE power sensor and an accelerometer, the signals were captured and utilized to identify the wheel condition and its level of wear. It was observed that when the GW began to wear out, the power signal exhibited an increasing trend. This indicates an increase in consumption for the same amount of material removed.

More advanced diagnostic methods have been developed with the assistance of AI. Wang et al. [172] introduced an intelligent decision-making system using power signals and found that their enhanced ANN algorithm improved prediction accuracy compared to a standard ANN. Zhang et al. [177] developed a wheel wear monitoring model based on a type-2 FNN. They claim that most prediction techniques used in grinding are valid only for specific materials and parameters. Therefore, the contribution of their work focused on establishing robust monitoring without the mentioned restrictions and better generalization capacity. Sauter et al. [178] developed a new method for detecting grinding burn with up to 99% accuracy. They measured in-process spindle current and power signals, then applied time–frequency transforms to extract statistical features for ML algorithms.

#### Summary of the Subsection

In this section, it was demonstrated how current and power sensors can be used in the monitoring and prediction of the GP behavior. It became evident that AI techniques have been increasingly gaining prominence in this area. Table 4 summarizes the cited studies.

**Table 4:** Methods of monitoring the GP using current and power sensors.

**Grinding Process**	**Year**	**Main application**	**Sensors**	**Reference**
GW	2021	Monitoring the grinding state of diamond rollers	HE current sensor	[171]
Cylindrical Grinding	2021	Identification of wheel wear	Power cell	[118]
Internal Plunge Grinding	2019	Improve the process grinding efficiency	Power sensor	[173]
Cylindrical Grinding	2022	Estimation of the grinding stock	Invertor	[175]
GW	2022	Understanding the impact of disc dressing parameters on grinding performance	Sensors in the CNC machine	[174]
GW	2023	Development of an innovative exponential polynomial model for surface roughness, linked to wheel conditions as indicated by cutting power	Power meter	[172]
GW	2021	Measurement of non-grinding power	HE power sensor	[176]
Cylindrical Traverse Grinding	2020	Development of a robust GW wear monitoring system	Power meter	[177]
GW	2021	Grinding burn detection	Power meter	[178]

### Vibration measurement sensors

During grinding, vibrations may appear in the system due to different sources, those related to the GW resulting from its unbalance or due to the progressive tool wear as machining takes place [150]. As machining progresses, the GW wears down, resulting in higher cutting efforts that cause vibrations in the system [58].

#### System overview

Vibrations are harmful to the process since their occurrence induces the generation of components with a worse finish (higher roughness values and irregular surface texture) and lower dimensional accuracy [179]. It is noteworthy that the natural vibration frequency of abrasive GWs is > 500 Hz or ideally > 1000 Hz, that is, vibration control is done by capturing the frequency during grinding [37]. Therefore, it is essential to monitor the system's vibrations, as the results can be linked to the condition of the abrasive GW. The primary sensor used for this purpose is the accelerometer. Normally, it is positioned close to the cut zone, as shown by Mahata et al. [118] in Fig. 19, and it captures the vibration signals, in this case in three orthogonal directions. After capturing the signals, it is necessary to use an (A/D) board, low-pass filter and a computational software for signal analysis [130].

As advantages of the accelerometer, low cost and ease of use stand out. However, Bahr et al. [129] highlights that the use of the accelerometer presents some challenges, such as the fact that the signal amplitude decreases with the distance from the cut region, but if the assembly is done very close, there will be great variability of the signals. As demonstrated by Samoylova et al. [180] in Fig. 20, the vibration signals generated during grinding are influenced according to the conditioning of the abrasive GW.

#### Applications in conventional and AI-driven Contexts

Several studies have been developed in order to understand the generation of vibrations during the GP and how their analysis can be useful for detecting the monitoring of the conditioning of the GW. Furthermore, it is noteworthy that some studies have also used AI in these analyses, contributing even more to the development of this line of research. In grinding tests on tempered steel, Hassui et al. [181] evaluated several output variables (roughness, vibration and AE) in order to understand the ideal moment for dressing the abrasive GW, that is, to observe the development of wear of the GW. The authors point out that the vibration sensor was the best in detecting the conditioning of the abrasive wheel, that is, in verifying the end of the abrasive tool's useful life. Thus, the RMS signal showed greater efficiency than the AE for this functionality. In another study developed by Hassui and Diniz [182], the authors also found a good correlation between the vibration signals, obtained by means of an accelerometer, with the conditioning of the abrasive GW, thus indicating the best time for dressing it.

Alfares and Elsharkawy [183] simulated grinding machine spindle vibration using a five degrees of freedom model in order to understand the correlation of abrasive GW wear on system vibration. The authors reported that the increase in abrasive tool wear resulted in an increase in vibration for all degrees of freedom. Thus, it appears that there is an acceptable wear limit and that after this value is reached, it is necessary to carry out the dressing operation of the GW in order to give the tool again cutting capacity. From experimental microgrinding tests, Feng et al. [127] studied the relationship between the conditioning of the abrasive GW with the output variables force (dynamometer), vibration (accelerometer) and acoustic emission (AE sensor). The authors further stated that although monitoring the conditioning of the abrasive wheel is complex, the fusion of vibration and force results developed during grinding makes it possible to monitor the wear of the abrasive tool. Xu et al. [123] developed a methodology for recognizing the abrasive wheel wear signature through energy decomposition using WPD for the GP. The authors emphasize that the results obtained indicated that the energy ratio removed from the vibration signals was related to the conditioning of the abrasive GW, that is, through this analysis it was possible to observe the level of wear of the abrasive tool.

Wang et al. [184] investigated the impact of force-controlled robotic grinding on the stiffness of the system and the surface finish of large thin-wall workpieces. The study highlights that the inherent low stiffness of thin-wall parts poses significant challenges due to vibration-induced instability during grinding, which adversely affects surface quality. By integrating a novel force-control end-effector into a robotic grinding workcell, the authors demonstrate that the system's dynamic characteristics can be altered to increase equivalent mass and damping, effectively suppressing vibrations. This vibration suppression mechanism improved grinding stability, allowing for deeper material removal per pass and enhancing the surface finish. Experimental results showed a significant improvement in surface roughness, with the average roughness (Ra) reduced from 2.457 μm to 0.762 μm, while maintaining stable grinding even under high grinding forces. The study underscores the role of force control in mitigating the adverse effects of low stiffness, enabling precise machining and superior surface quality in robotic grinding applications.

Dong et al. [185] studied the impact of handheld workpiece vibration during GPs, with a particular focus on system stiffness and its influence on surface finish. The study developed a comprehensive model of the grinding machine-workpiece-hand-arm system to analyze vibration responses and evaluate engineering methods for vibration control. Results revealed that the dynamic stiffness of the grinding interface plays a critical role in determining the vibration behavior and surface quality of the workpiece. A reduction in interface stiffness effectively lowers vibration amplitudes, particularly in the resonant frequency range, leading to improved surface finish. The study also highlighted that high-frequency vibrations, while challenging to control, can be mitigated through methods such as vibration absorbers and adapters, further enhancing the quality of the machined surface. These findings underscore the importance of optimizing system stiffness and vibration control strategies to achieve superior surface finishes in handheld workpiece grinding.

As already shown by other studies already listed, the conditioning of the GW deserves attention since its wear can cause system vibrations. Therefore, the dressing operation is used for this function. However, the dresser can also wear out, which will impact the conditioning of the abrasive tool. Thus, it is important to change the dresser when it is not able to give the GW the necessary conditions. In this sense, Miranda et al. [131] sought to analyze the signals of vibration and AE to determine the wear of the dresser using fuzzy models. The results obtained showed that this adopted methodology showed high efficiency in predicting the wear of the dresser. D’Addona et al. [126] acquired vibration signals and applied ANNs to understand the conditioning of the abrasive GW during the GP. The authors observed that ANN processing proved to be effective in monitoring the conditioning of the abrasive GW through the analysis of vibration signals from the system. Thus, from the analysis of the vibration arising during machining, it is possible to understand the wear rate of the abrasive GW. In research developed by Mahata et al. [118] was presented a cost-effective methodology for monitoring GW wear in cylindrical grinding using inexpensive sensors like accelerometers and power cells. Vibration and power signals are processed with the HHT to extract key features, such as the Hilbert Spectrum Centroid (HSC), which distinguish between sharp and worn-out wheel states. Fig. 21 illustrates the systematic workflow, including signal acquisition, feature extraction, and classification using Support Vector Machines (SVM), achieving 100% accuracy across different cutting depths. The study highlights the robustness of the method, its potential for optimizing dressing intervals, and its practicality for real-time industrial applications.

#### Summary of the Subsection

During grinding, the GW wears out, causing an increase in cutting forces and, consequently, causing vibrations in the system. These vibrations are harmful to the process as they provide the generation of components with a worse finish and greater dimensional deviations. In this sense, it is important to monitor them since several studies have indicated a direct relationship between abrasive tool wear and vibration signals. In addition, AI has also been used in these analyses, seeking to develop reliable and robust methodologies for monitoring abrasive GW conditioning. In Table 5 are shown the advantages and limitations of each of these sensors.

**Table 5:** Tool monitoring methods for grinding.

**Grinding Process**	**Year**	**Main application**	**Sensors**	**Reference**
Plunge cylindrical grinding	1998	Tool wear diagnosis	Vibration sensor and AE sensor	[181]
External cylindrical grinding	2000	Tool wear diagnosis	Power sensor and AE sensor	[123]
Cylindrical grinding	2001	Tool wear diagnosis	Force sensor, vibration sensor and AE sensor	[98]
Plunge cylindrical grinding	2003	Surface roughness diagnosis, Tool wear diagnosis	Vibration sensor	[182]
Surface plunge grinding	2004	Tool wear diagnosis	Force sensor	[159]
Surface grinding	2006	Tool wear diagnosis	Force sensor	[114]
Cylindrical grinding	2008	Tool wear diagnosis	Power sensor, vibration sensor and AE sensor	[124]
Microgrinding	2009	Tool wear diagnosis	Force sensor, vibration sensor and AE sensor	[127]
Cylindrical grinding	2010	Tool wear diagnosis	Vibration sensor	[186]
Microgrinding	2014	Tool wear diagnosis	Force sensor	[125]
Surface grinding	2017	Tool wear diagnosis	Power sensor	[145]
Abrasive belt grinding	2018	Surface roughness diagnosis, Tool wear diagnosis	Force sensor	[15]
Cylindrical grinding	2018	Tool wear diagnosis	AE sensor	[146]
Surface grinding	2018	Surface roughness diagnosis, Tool wear diagnosis	Force sensor	[162]
Surface grinding	2019	Tool wear diagnosis	Force sensor, vibration sensor and AE sensor	[147]
Cylindrical traverse grinding	2020	Tool wear diagnosis	Power sensor, vibration sensor and AE sensor	[148]
Cylindrical grinding	2021	Tool wear diagnosis	Vibration sensor and power sensor	[118]

### Acoustic emission detection sensors

During grinding, AEs are generated as high-frequency sound waves due to the interaction between the GW and the workpiece [187]. These emissions can originate from various sources, such as micro-cracking, plastic deformation, and friction at the contact zone [188]. As the GW wears or experiences changes in its condition, the characteristics of the AEs alter, providing valuable insights into the tool's health and the GP efficiency [189].

#### System overview

**Fig. 22:**
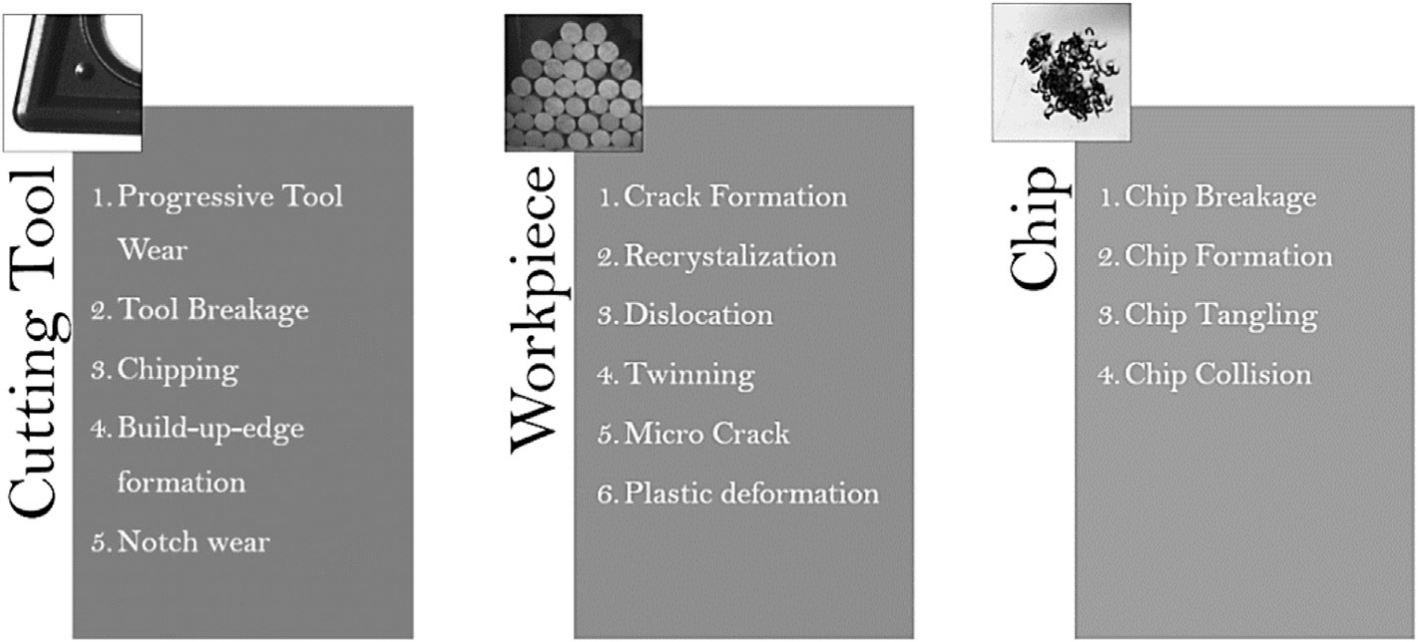
Primary sources of AE formation.

The recognition of the cutting tool wear in random time heavily depends on the ability to monitoring of the cutting zone with various sensor systems. AE sensors were developed for these kinds of detections different from the sound sensors or microphones as they can sense the high frequency waves [190]. AE means the elastic wave generation in the material which exposes an external force [191]. From the above-mentioned information, using an AE sensor aims to predict progressive tool wear, various types of tool wear and tool breakage primarily. However, the sensor is able to discover the changes depend on chip movements such as tangling and breaking [192]. Broad number of material activity such as crack propagation, twinning, shearing of crystal plane and martensitic phase transformation trigger the AE signal waves. Basically, chip, workpiece and cutting tool dependent events affect the AE signal fluctuations. In this respect, the possible sources of AE signal waves are schematically represented in the Fig. 22.

During regular chip removal, cutting operations produce continuous signals. However, when an extraordinary event occurs, the signal becomes burst-type [193]. Researchers classify AE sources into two main categories: macroscopic sources, which are relatively severe events like breakage, collision, and fracture, and microscopic sources, which are relatively minor events such as dislocation movement and void nucleation [194].

With its extensive incident-capturing and distinguishing capability, the sensor has been widely used in the past due to its high price/performance ratio and convenience for remote sensing and online evaluation [195]. AE sensors excel in monitoring systems with specifications like good material anisotropy and less sensitivity to geometrical changes. Despite these advantages, there are some restrictions: AE waves are weak signals, making them difficult to capture; AE sensors are highly sensitive to foreign sounds, causing signal disorder; and there is low repeatability of sensor signals due to the excessive number of combinations of AE sources [196].

**Fig. 23:**
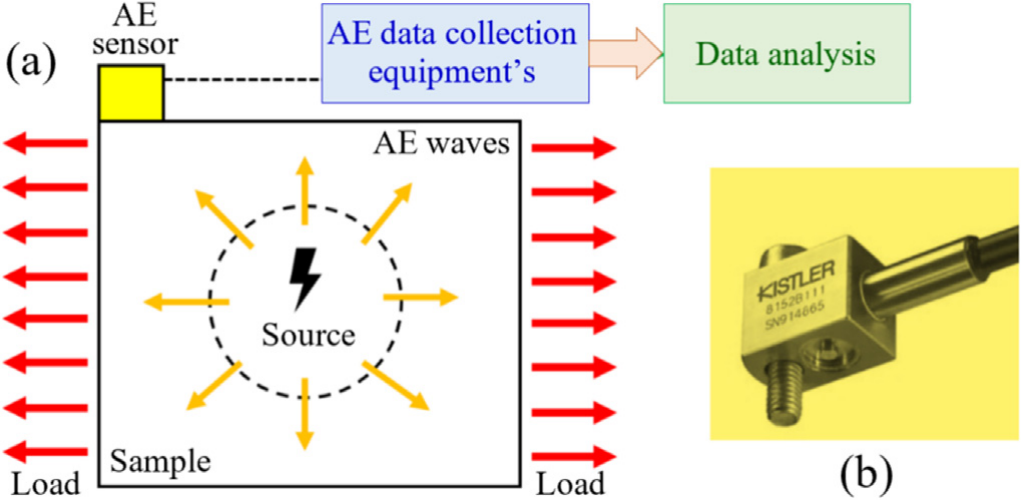
Working principle of AE sensor (a) and an example for AE sensor used in condition monitoring (b).

A schematic representation of the working principle of AE sensor and a photo of an actual sensor is presented in Fig. 23. As showed in Fig. 23a, an AE sensor can be placed to a ground with mechanical connection, otherwise the link can be provided magnetically. Therefore, the sensor can be fixed several points on the machine tool such as carriage, toolholder and workpiece etc. depending on the operation type and machine tool specifications.

#### Applications in conventional and AI-driven Contexts

Grinding is a precision engineering abrasive-finishing process primarily used for hard materials. The grinding tool, with its numerous grits around the wheel, performs continuous cutting operations. Since grinding is the final manufacturing step, minimizing errors is crucial to reduce costs, labor, waste, and improve productivity. Therefore, monitoring the GW is essential. AE sensors have become a popular choice for TCM due to their ease of implementation, sensitivity, and relatively low cost. As described in the previous section, AE signal development is influenced by interactions between the tool, workpiece, and chip. GW has numerous grains, each one can be considered as micro-cutter which is a potential AE source [197]. Especially thinking on the cutting of hard materials, high-strength property of the work material causes rapid changes on the grains. Therefore, it is expected to observe intense plastic deformation on the workpiece surface. In addition, many interactive events due to grains and their functions occur such as fracture, ploughing and rubbing etc.

From the above-mentioned literature analysis, researchers have been profited by AE sensors on grinding operation in the last years. For example, Han and Wu [198] examined AE signals to find a relationship between GP for high precision. Accordingly, there was a strong relation between AE signals and machining process. Moreover, different grinding directions had influence on AE signals, also the authors recommended a distance (<80 mm) between the AE source and transducer for high sensitivity. The intensity of the contact conditions heavily depends on the abrasive grains which can be correlated with the AE signals. Life cycle assessment of the GW was carried out by an AE sensor while providing 90% accuracy during grinding [199]. The authors used neural networks and regression model to estimate the wheel life by applying AE sensor signal features.

**Fig. 24:**
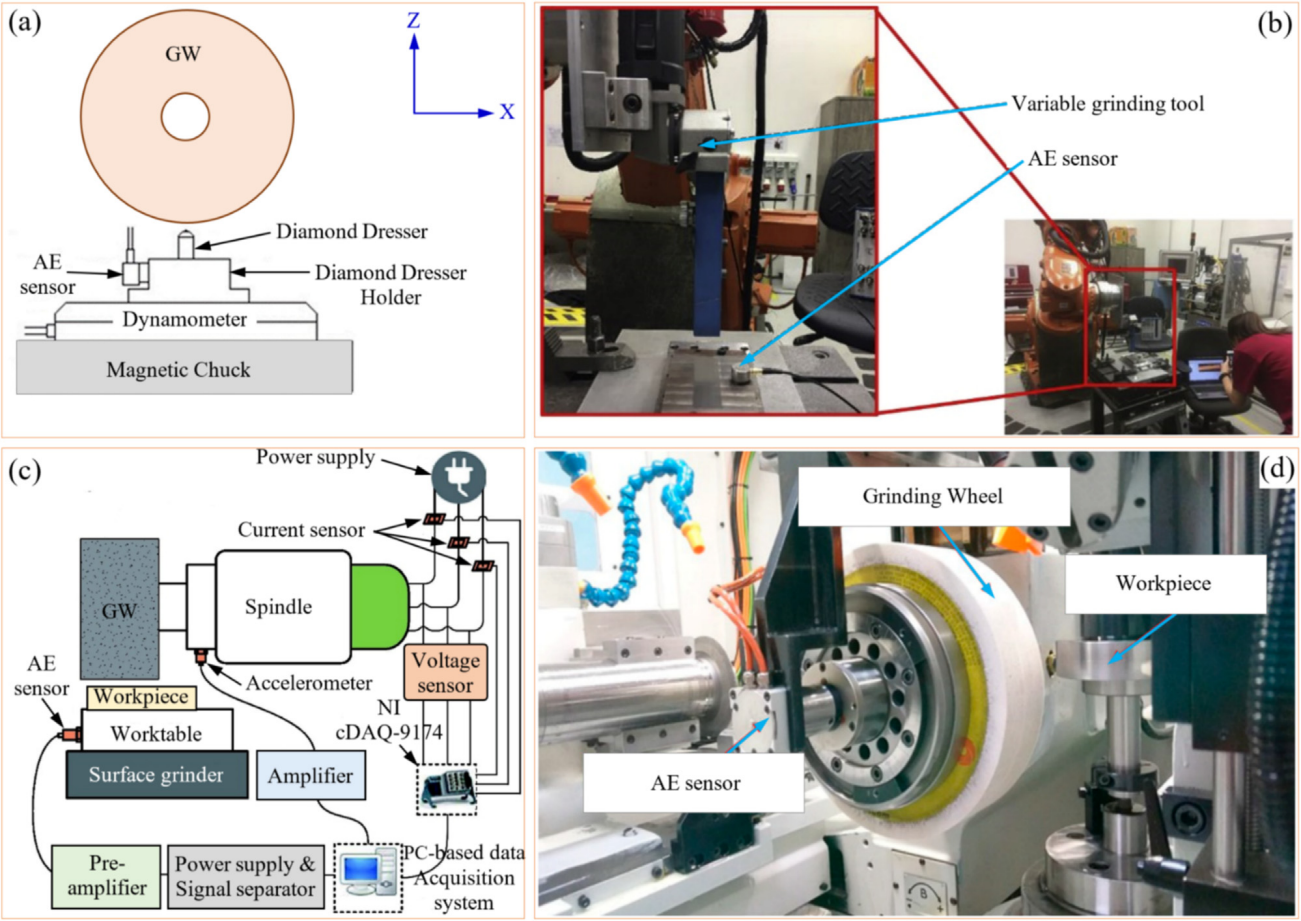
Some applications of AE sensor on grinding operations placed on clamp (a) [193], workpiece (b) [191], worktable (c) [205] and spindle of GW (d) [163].

Dias et al. [200] carried out an approach for detection and prediction of surface roughness, roundness and cylindricality with the help of AE signals. Bhuiyan et al. [201] detected different ranges of AE signals for determination of tool wear and plastic deformation events. An effective method was proposed to differentiate these two facts. Based on SVM, Zhang et al. [202] proposed monitoring study using AE sensor for improving the surface roughness during grinding and over 75% accuracy was obtained according to the results. Liao [203] proposed a method based on feature extraction and selection for condition monitoring with AE sensor. Best features were determined for posing of the GW. For grinding burn detection, Chen and Gindy [204] investigated AE features which were found as an efficient method for this purpose. Badger et al. [193] evaluated the AE signals on measuring the influence of different grit modes with describing a new AE energy parameter. The experimental setup is demonstrated in Fig. 24a and the proposed method was accepted as effective for industrial work. Pandiyan and Tjahjowidodo [191] used AE sensor to examine the contact conditions as a result of tool wear during grinding as represented in Fig. 24b. Yang et al. [205] evaluated the performance of AE sensor on detection capability of grinding burn which is accepted as one of the major limitations in precision grinding as represented in Fig. 24c. Promising results were obtained with the proposed method that uses HHT as signal processing tool.

The paper by Liu et al. [163] proposed a novel measurement method for monitoring GW loading in continuous generating gear grinding machines using an AE sensor, as illustrated in Fig. 24d. By analyzing AE signals, the surface roughness of ground workpieces was evaluated using a digital image processing technique, enabling the assessment of machining quality. This approach effectively correlates AE data with GW loading and surface roughness, providing an early warning system for tool wear and maintaining high machining precision. Similar studies, such as those by Krishnan and Rameshkumar [206], have utilized AI models like the Hidden-Markov model to predict AE data, achieving high accuracy. The integration of AE sensors and AI techniques demonstrates significant potential for improving GPs by enabling precise monitoring, prediction, and optimization of tool wear and surface finish.

Wang et al. [207] presented a novel approach for identifying GW wear states using AE signals, the HHT, and the RF algorithm. AE signals, highly sensitive to grinding conditions, are processed using the Empirical Mode Decomposition (EMD) method to extract intrinsic mode functions (IMFs) with strong correlation to the original signal. Key features such as maximum value, RMS, and spectral centroid are calculated from the Hilbert marginal spectrum of selected IMFs. These features are optimized using RF-based feature importance and out-of-bag error analysis, enabling accurate classification of GW wear into mild, medium, and severe states with a comprehensive accuracy of 93.3%. The proposed method demonstrates its effectiveness in real-time monitoring, providing a robust and adaptive solution for precision grinding applications.

Seemingly, there is a motive for using AE sensors during grinding of the various types of materials such as carbon steels, aluminum alloys and hardened steels for surface finishing. According to the author’s reports, grinding is an authorized method for these types of operations to improve the surface quality where AE signals have superior properties to monitor the cutting area. In addition, AE signals are good indicators to represent the health of the GW on different times of cutting operations. Moreover, AE sensors are competent for positioning of the GWs to produce high precision surfaces. It was reported that many AI approaches have been used for signal processing of AE data and that indicates the compatibility of these signals to be processed via different ways. There is an agreement that AE sensors have good price/performance rate which makes them preferable.

#### Summary of the Subsection

Use of AE sensors have became widespread with the increasing expectations from the machining sector namely minimum mistake, cost and maximum performance and reliability. As an easily fastened and applicable sensor AE is an effective tool especially in detecting tool breakage compared to other types of sensors used in TCM. Not only for tool status, but also for workpiece condition and chip forming norms can be effectively controlled by the AE technology which highly depends on the sensitivity range. Main disadvantage of the sensor is the repeatability of the machining test because of the external/internal sources can be easily penetrate to the signal waves and deteriorates the signal structure. This is interestingly an advantage in other way which provides to catch many microscopic and macroscopic actions realized around the machining environment. Table 6 provides a summary of the referenced studies.

**Table 6:** Methods of monitoring the GP using AE sensors.

**Grinding Process**	**Year**	**Main application**	**Sensors**	**Reference**
Surface grinding	2010	Detecting tool wear and plastic deformation events	AE sensor	[212]
Cylindrical grinding	2011	Monitoring surface roughness, roundness, and cylindricality	AE sensor	[211]
Surface grinding	2013	Improving surface roughness using SVM-based monitoring	AE sensor	[213]
Surface grinding	2014	Feature extraction for condition monitoring	AE sensor	[214]
Surface grinding	2015	Burn detection using AE features	AE sensor	[215]
Plunge grinding	2015	Evaluating influence of grit modes with AE energy parameter	AE sensor	[206]
Cylindrical grinding	2016	Contact condition monitoring due to tool wear	AE sensor	[204]
Surface grinding	2017	Detecting grinding burn using HHT	AE sensor	[216]
Gear grinding	2020	GW loading and surface roughness monitoring using AE signals	AE sensor	[118]
Surface grinding	2022	Identifying GW wear states using RF and HHT	AE sensor	[218]
Surface grinding	2010	Detecting tool wear and plastic deformation events	AE sensor	[212]
Cylindrical grinding	2011	Monitoring surface roughness, roundness, and cylindricality	AE sensor	[211]
Surface grinding	2013	Improving surface roughness using SVM-based monitoring	AE sensor	[213]
Surface grinding	2014	Feature extraction for condition monitoring	AE sensor	[214]

### Image sensors

The main advantage of image sensing lies in the fact that it does not interfere with the GP and consequently change its actual behavior. Furthermore, there is no need to remove the tool, which promotes fast and cost-effective measurement. However, new developments in the field are necessary to make this method more robust in an industrial environment, as debris can adhere to the lenses and compromise the system [208].

#### System overview

**Fig. 25:**
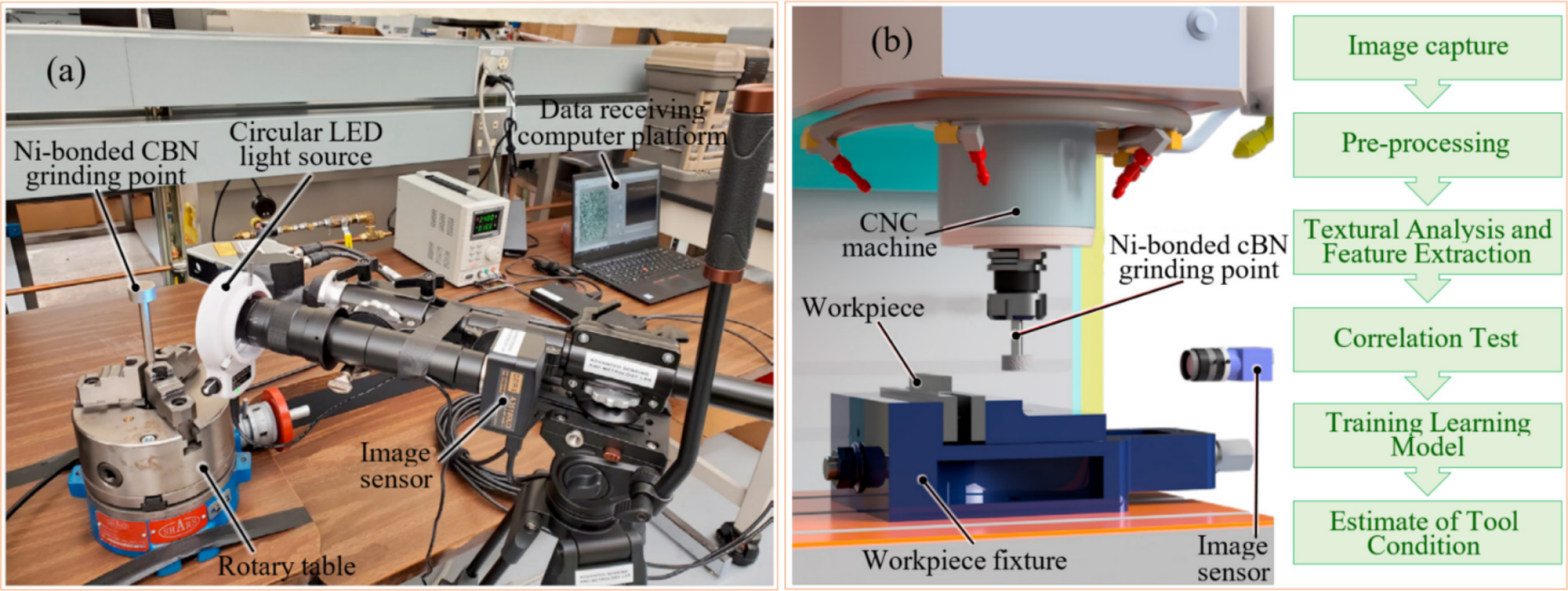
Example of a sensor system (a) and its application with all essential stages (b) [209].

The sensor system generally consists of a CCD camera, a light source, and a computer platform. An illustrative example is depicted in Fig. 25. The image sensor, mounted on a tripod, captures the perpendicular cross-section of the GW's surface. A ring-shaped LED (light-emitting diode) light provides stable illumination, while a customized OMEX CCD camera captures surface images around the cBN (cubic boron nitride) GW's circumference.

The image processing phase involves modifying and preparing pixel values to create images suitable for further operations. Steps include converting images to grayscale to simplify processing, analyzing texture to extract statistical features, evaluating and selecting highly correlated features, training a neural network, and estimating the GW's condition [209]. Depending on the image quality, noise suppression and image segmentation are also included [82].

Key statistical characteristics include mean, standard deviation, kurtosis, skewness, and entropy. The mean indicates light intensity, with higher values signifying more wear in black bonding material. Standard deviation measures grain distribution variance. Skewness, either negative or positive, indicates symmetry and grayscale intensity distribution. Kurtosis assesses pixel clustering at similar grayscale levels, with higher values suggesting flatter grains due to wear. Entropy measures the randomness of grayscale distribution, reflecting the geometric patterns of grains with new cutting edges. These features collectively represent the physical characteristics of the grains' cutting edges and planes.

Su and Tarng [72] emphasize key considerations for using image sensing. Firstly, CCD camera images may have focus issues. Secondly, proper lighting techniques are crucial to improve contrast and distinguish the measured object. Lastly, the vision system requires calibration to ensure accurate measurements, addressing camera distortion, perspective errors, and spatial calibration.

#### Applications in conventional and AI-driven Contexts

Wheel wear level is a crucial parameter in the field of grinding as it can significantly impact the efficiency of the manufacturing process. A notable trend observed in recent articles is the development of correlations between the image of the GW and various operational parameters. Lee et al. [209] introduced a method for estimating the tool condition of a cBN GW using an image sensor. These images were subsequently utilized in ML techniques, specifically ANNs, to establish predictive equations, achieving a coefficient of determination (R^2^) of 0.959. Liu and Ou [210] utilized digital image processing techniques to identify the loading area on Al_2_O_3_ GWs. This allowed them to isolate regions with metal loading debris and calculate the corresponding loading percentage.

**Fig. 26:**
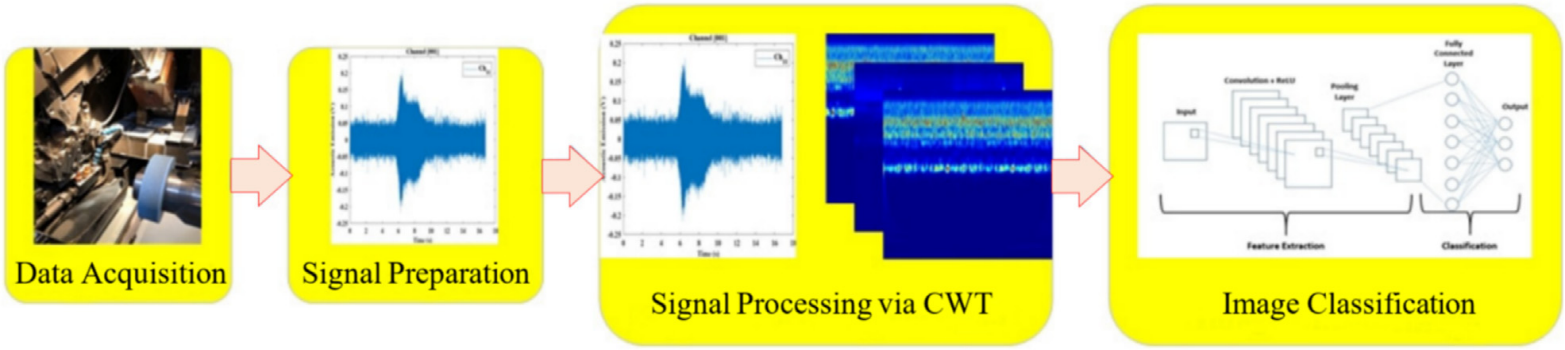
Procedure for image classification of grinding burns based on AE signals [211].

AE sensoring is another method for monitoring grinding. Ceylan et al. [211] proposed the conversion of these signals into scalogram images and subsequently classified the type of grinding burn using pre-trained neural networks. Fig. 26 outlines the adopted procedure. CWT stands for Continuous Wavelet Transform.

Wang et al. [212] state that in certain studies related to monitoring techniques, the model's performance is influenced by grinding parameters. To address this problem, they developed an abrasive wear state classifier utilizing surface images and the RF algorithm. This approach achieved a 99% accuracy rate in identifying belt wear states, effectively reducing the model's dependency and sensitivity to changes in process parameters.

For monitoring the MRR during belt grinding, a multi-sensor fusion approach that integrates vision and sound can be employed. Wang et al. [213] demonstrated this method, which achieved an error rate of only 3% using the enhanced gradient boosting machine algorithm. The grinding spark images were analyzed based on color and texture features.

#### Summary of the Subsection

In this section, various approaches involving the application of image sensors for monitoring the GP have been presented. AI, primarily used for classification purposes, can be implemented through different techniques. Table 7 provides a summary of the referenced studies.

**Table 7:** Methods of monitoring the GP using image sensors.

**Grinding Process**	**Year**	**Main application**	**Sensors**	**Reference**
GW	2021	GW wear	CCD camera	[209]
GW	2007	GW wear	CCD camera	[82]
GW	2006	GW wear	CCD camera	[72]
GW	2020	Loading evaluation	CCD camera	[210]
GW	2023	Classification of grinding burns	AE signal converted into image	[211]
Belt grinding	2022	Monitoring of belt wear state	CCD camera	[212]
Belt grinding	2021	MRR monitoring	CCD camera	[213]

### Other sensors (Temperature, Ultrasonic, Optical, and Laser)

#### System overview

**Fig. 27:**
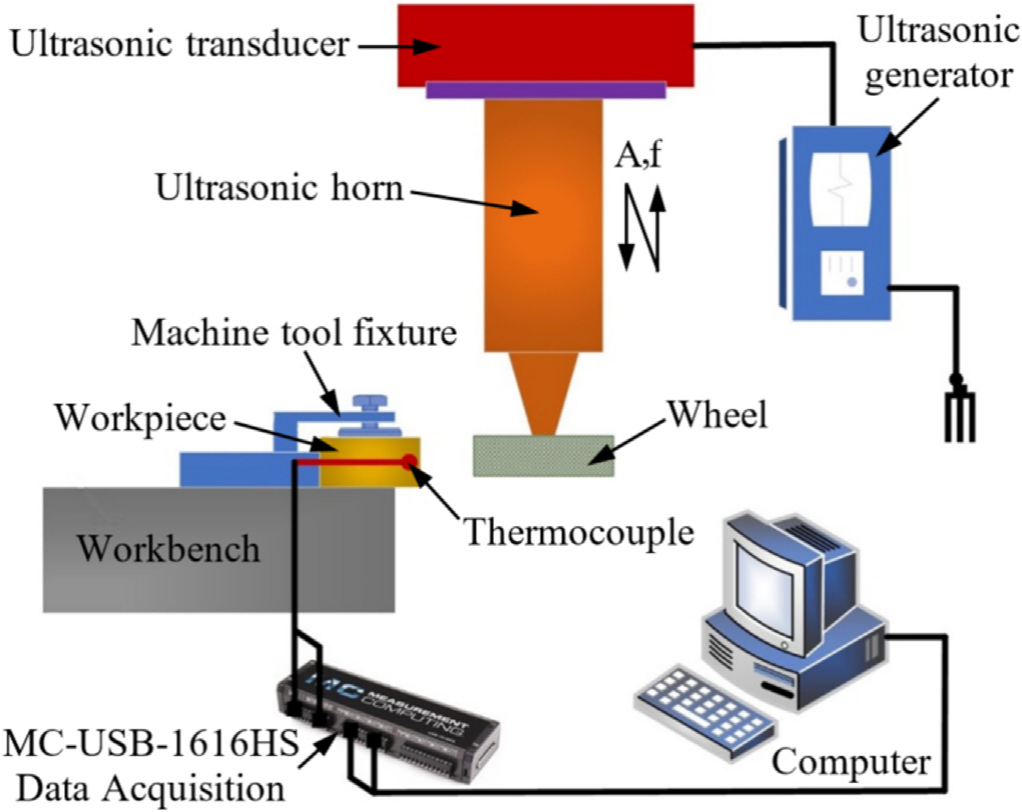
Experimental ultrasonic vibration grinding [215].

In order to carry out an experimental grinding study, firstly it is necessary to create an experimental grinding environment. In addition, an expert and special tools and equipment are needed in this regard. Fig. 27 shows the experimental setup for ultrasonic vibration grinding. However, it also requires a lot of time and cost. AI methods, especially ANN, can be solved very easily for complex, non-linear problems that cannot be mathematically modeled or solved with classical methods, and are used very successfully by eliminating such limitations and inadequacies in the conventional systems [214].

#### Applications in conventional and AI-driven Contexts

Learning in a neural network involves finding the optimal values for all connection weights in the ANN, allowing it to produce the correct outputs for a given set of input and output data [135]. This process continues until the error between the predicted output and the desired output falls below a certain value or the training process reaches a certain number of repetitions [136]. Therefore, the learned information is stored on the connecting lines between the processing elements and displayed by means of weights. These weights can be thought of as elements that store certain features of the related problem in memory. Information processing, on the other hand, can be interpreted as recalling relevant features from memory when an event is shown and making decisions by analyzing the inputs related to them together [137]. Since the processing elements in the input and output layers are known, the best performance of the ANN, that is, the optimum or near-optimal hidden layers that make the network error minimum and the learning speed maximum, and the number of processing elements in each hidden layer are determined by trial and error [138]. Too few processing elements reduce the learning rate, while too many processing elements or hidden layers slow down or in some cases make learning difficult. Generally, a training algorithm is used for the learning process and how the weights will be arranged according to a learning sample is determined by this algorithm. In the first step of the training process, which training algorithm will be used is specified in the ANN studies [216]. By making arrangements for the number of hidden layers to be one or more; the number of input, hidden and output layer processing elements is adapted. After the number of layers is entered, the number of iterations is entered by the user and the training process starts. Training continues until the iteration is finished or the desired error amount is reached [217]. At the end of the training process, the representative ANN model results corresponding to the analytical calculation results for both the training and the test set. The implementing processes of the ANN was repeated by changing different training algorithms, network structure by trial and error [218]. Analytical calculation results and ANN model results are compared with statistical error analysis. In statistical error analysis, the performance of the training set and the test set were evaluated together [219].

#### Summary of the Subsection

**Fig. 28:**
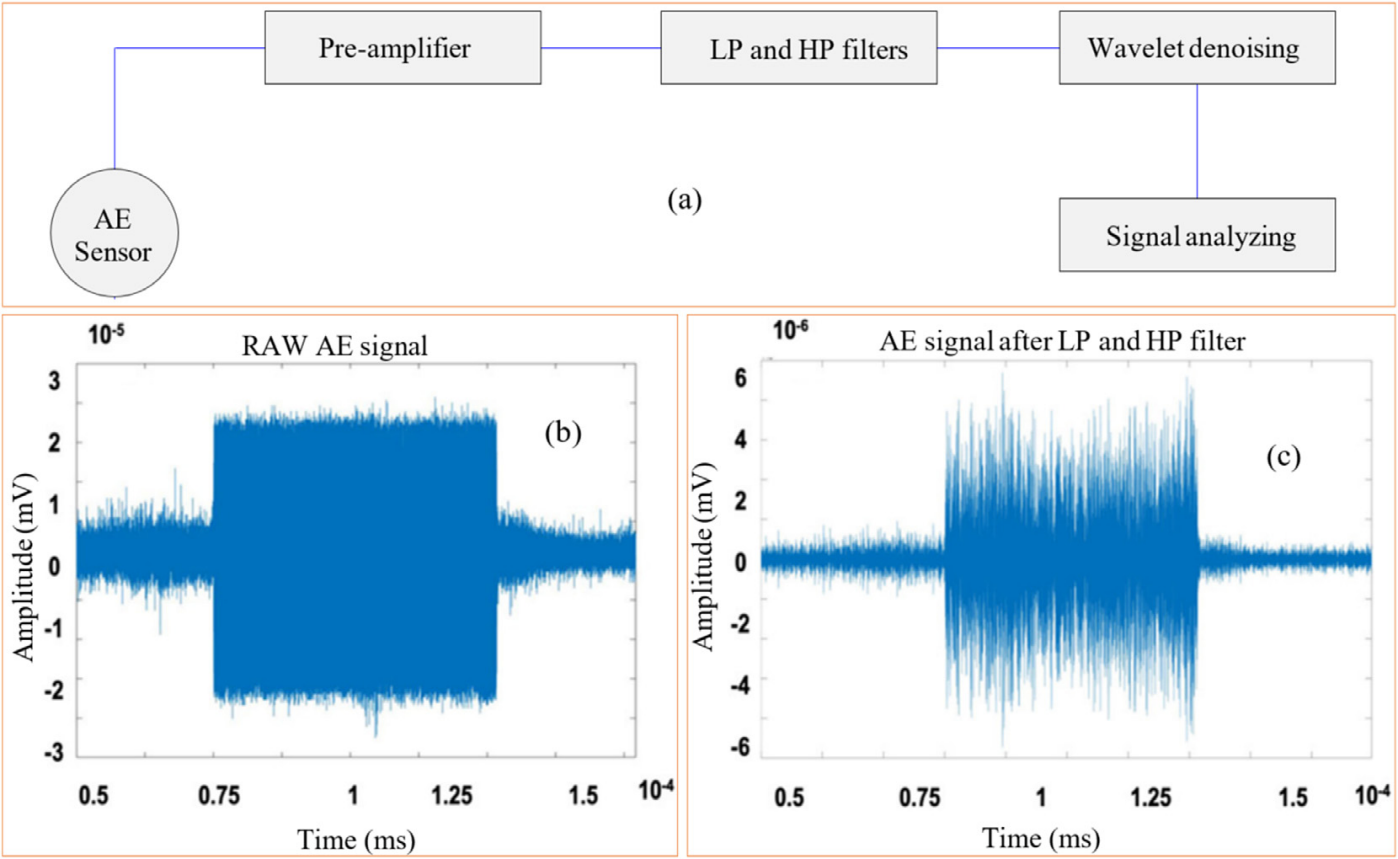
AE filtering process (a), Raw acoustic signal (b) and AE signal post Low Pass (LP) and High Pass (HP) filtering (c) [221].

The importance of AE sensors lies in their ability to monitor, control, and predict machining processes [123]. As a result, their application is growing in numerous scientific and industrial sectors. It has been shown that the AE signal produced during grinding is connected to the grinding tool, the process state, and the surface condition (Fig. 28) [220].

Using this method, researchers can improve various aspects of machining, including wheel wear and load detection, vibration analysis, collision detection, eliminating gaps in the detection of burials and cracks, process control, dressing, and more [193]. An ANN is a computing system inspired by its biological counterpart. These systems learn to perform tasks without explicit programming and are highly effective in ML, particularly in research and development.

The industry seeks higher productivity, reduced costs, improved quality of crushed products, online condition monitoring, and process feedback control [222]. Research on the GP often focuses on modeling to understand material removal mechanisms, especially single-grain workpiece interactions [223], [224]. Additionally, microscale numerical modeling techniques have been developed to describe the plastic behavior of materials at high temperatures and distortion rates during grinding [225]. Table 8 provides a summary of the referenced studies.

**Table 8:** Tool monitoring methods for grinding.

**Grinding Process**	**Year**	**Main application**	**Sensors**	**Reference**
Grinding	1964	Changing behaviour of cutting edges	Optical sensors	[226]
Abrasive finishing	2004	Contact detection	Current and power sensors	[104]
Grinding	2007	Condition of GWs	Optical sensors	[82]
Grinding	2007	Assessment of GWs	Optical sensors	[227]
Grinding	2013	Grinding burn	Current and power sensors	[228]
Grinding	2013	Description of GW topography	Optical sensors	[229]
Cylindrical Grinding	2018	Condition of grinding tools	AE Sensor	[146]
Grinding	2018	Estimation of GWs life	Multi sensor	[230]
Abrasive belt grinding	2019	Assessment of Surface quality	Optical sensors	[231]
Grinding	2021	Condition of GWs	Image sensor	[232]
Grinding	2020	Grinding condition factors	Wireless sensor	[233]

### Temperature sensors

**Fig. 29:**
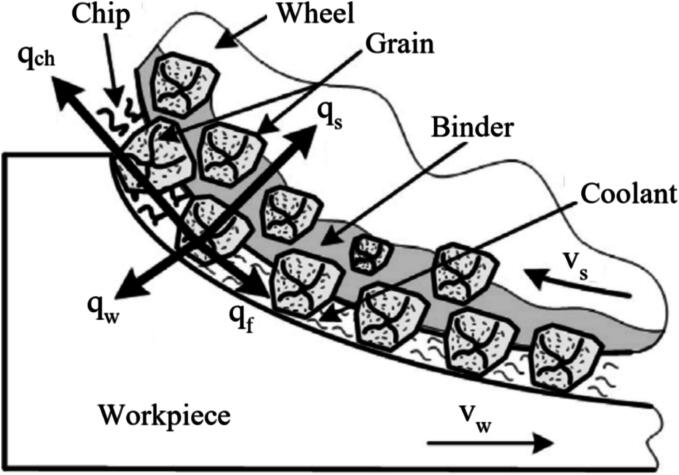
Contact zone heat transfer paths [234].

Fig. 29 ilustrates that the heat from friction at the abrasive-workpiece interface dissipates in four directions: into the workpiece ($q_{w})$, the tool ($q_{s})$, the cooling fluid ($q_{f})$ and the chips ($q_{\mathrm{ch}})$. The GW speed is denoted by $v_{s}$, and the workpiece speed by $v_{w}$.

Table 9 summarizes various techniques. Some methods are more cost-effective but lack precision, while others provide acceptable results but may not be economically viable. Typically, grinding temperature is measured using TCs and infrared radiation (IR) [235].

**Table 9:** Pros and cons of various methods for temperature measurement.

Technique	Key Benefits	Key Drawbacks	Additional Notes
Thermal paint	Simple and budget-friendly	Inaccurate and error-prone	Should be verified with a more precise method
TC			
Tool-work	Easy to implement	Requires rearrangement, challenging calibration, slow response	
Transverse	Measures temperature at multiple points without setup changes	Not suitable for grinding, drilling, milling, etc.	
Embedded	Ideal for specific machining processes	Cannot measure surface temperature directly	
Pyrometer			
Infrared	Delivers quick results	Destructive, influenced by emissivity changes	Sensitive to ambient temperature and IR
Optical	Usable on any surface	Cannot plot complete isotherms for all materials	
Infrared photographic	Extremely fast, suitable for hazardous environments	Requires preheating, costly	Provides direct, long-lasting readings
Fine powder	Cost-effective	Unreliable, approximate results	No calibration required
Metallographic	Accurate within ± 25 °C in the 650–900 °C range	Limited to specific materials	Calibration needed

#### System overview

**Fig. 30:**
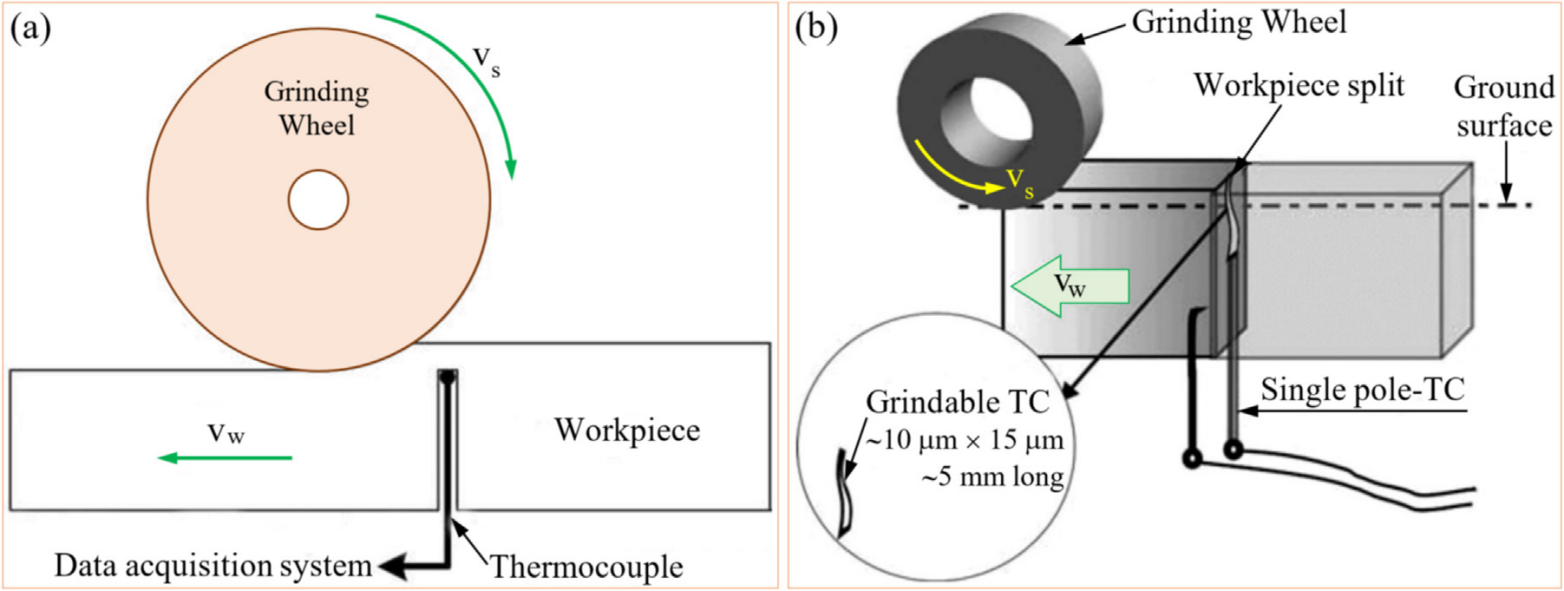
Configuration of embedded TCs (a) and foil-workpiece TCs (b).

Adapted from [236]

In their work, Liu et al. [236] highlight that TCs and infrared techniques are frequently employed and hold promise in the context of the GP. TCs are commonly utilized due to their ease of installation and cost-effectiveness in industrial applications. Common implementations include variations like embedded TCs and foil-workpiece TCs, as shown in Fig. 30.

In the embedded method, a TC is placed in a blind hole within the workpiece to measure grinding temperature, providing a robust temperature signal but often underestimating actual surface temperatures due to steep, nonlinear gradients near the surface. The foil-workpiece method, involving two split workpieces holding TC foils that form a junction during grinding, offers precise surface temperature measurement with rapid microsecond response time. However, it is sensitive to noise and may yield unreliable signals in wet grinding conditions.

**Fig. 31:**
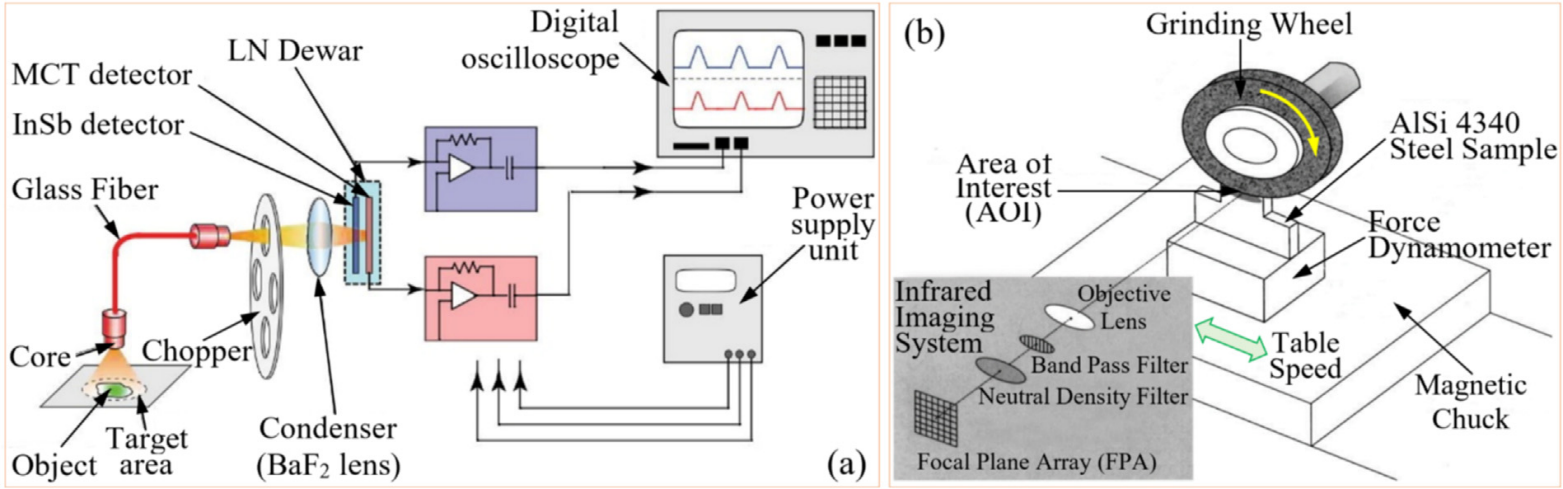
Configuration of infrared pyrometer (a) [236] and thermal imaging (b) [237].

Infrared radiation techniques measure grinding temperature by detecting IR emitted by the workpiece. This non-contact method minimizes interference from mechanical vibrations and includes two approaches: thermal imaging and infrared pyrometers. Fig. 31 provides a schematic illustration of these different variants.

Thermal imaging offers real-time temperature distribution visualization through an array of thermal camera sensors. However, this method encounters challenges when trying to measure temperatures within the contact zone impacted by grinding fluids. Additionally, its relatively low sampling rate limits its ability to capture rapid temperature fluctuations. Lastly, obtaining a high-quality thermal camera can be costly. On the other hand, the IR pyrometer with optical fiber boasts high spatial resolution and rapid response times. Nevertheless, it faces difficulties in directly measuring surface temperatures and setting up the measurement equipment can be intricate. Maintaining cleanliness and precision at the fiber ends is essential, making them susceptible to fragility under specific working conditions.

#### Applications in conventional and AI-driven Contexts

Recently, Liu et al. [238] introduced an innovative experimental approach for monitoring temperature at various points on the workpiece. They used an embedded array of bipolar TCs with welded wires, placed into through-holes and insulated with resin. As the GW passed over the microholes, numerous grains interacted with the workpiece, enabling the TCs to record temperature data. Miao et al. [239] employed semi-natural TCs constructed from constantan wire and the workpiece to assess temperature variations at different locations. This approach enabled them to pinpoint temperature extremes, including the root peaks and valleys, on a single-crystal nickel-based turbine blade.

To investigate temperature fluctuations under varying grinding conditions, Yan et al. [240] positioned a K-type TC 2 mm below the grinding surface on the side of the workpiece. This placement allowed them to monitor temperature changes during the GP. In a study by Jiang et al. [241] on quenched automotive transmission gears, TCs were strategically placed in narrow slots on the workpiece to measure grinding temperature. The tips were positioned near the grinding surface, and the slots were sealed with waterproof and heat-resistant adhesive to prevent interference from cooling liquids. Hou and Yao [242] used foil-workpiece TCs to study grinding temperature and heat flux distribution with grooved GWs. They designed K-type TCs with two parallel metal sheets, each 1 mm wide and 0.1 mm thick, to enhance measurement precision and stability.

Liu et al. [236] introduced an approach tailored for creep-feed grinding applications, emphasizing the examination of the workpiece's rear surface using infrared temperature mesurement. By employing a two-dimensional sliding table, they could precisely target the small hole within the workpiece, ensuring accurate measurement of the grinding temperature. This technique circumvents issues related to high-frequency signal interruptions stemming from mechanical vibrations and electrical interference, thanks to its non-contact nature and signal shielding. Shakouri and Mirfallah [96] utilized IRT to track thermal fluctuations in a bone during high-speed grinding.

Following temperature data acquisition, AI techniques can be leveraged to construct predictive models. In their work, Liu et al. [243] harnessed data collected from embedded TCs to develop a neural network model capable of forecasting high-speed grinding temperatures for titanium matrix composites. They compared different optimization algorithms and evaluated the effect of grinding parameters on temperature. Similarly, Mitrofanov et al. [244] used an ANN to predict temperature and cutting force while grinding a nickel alloy with minimal lubrication delivered by compressed cooled air. Experimental data was collected using embedded TCs, and their study found that the best predictions were achieved through this approach.

#### Summary of the Subsection

In this section, various approaches involving the application of temperature sensors for monitoring the GP have been presented. Table 10 provides a summary of the referenced studies.

**Table 10:** Methods of monitoring the GP using temperature sensors.

**Grinding Process**	**Year**	**Main application**	**Sensors**	**Reference**
GW	2023	Tribology	Embedded TC	[238]
GW	2023	Single crystal nickel base turbine blade	Wire-workpiece TC	[239]
GW	2023	Determination of temperature variation under different grinding parameters	Embedded TC	[240]
GW	2022	Quenched automotive transmission gear	Embedded TC	[241]
Grooved GW	2023	Grinding temperature and heat flux distribution	Foil-workpiece TC	[242]
GW	2016	Creep-feed grinding	Infrared thermometer	[236]
GW	2019	High-speed grinding of bone	IRT	[96]
Belt grinding	2022	Effect of grinding parameters on temperature	IRT	[245]
GW	2017	Titanium matrix composites	Embedded TC	[243]
GW	2021	Nickel alloy	Embedded TC	[244]

### Ultrasonic sensors

Ultrasound finds extensive use across various engineering applications, predominantly in non-destructive evaluations. Its functioning revolves around the manipulation of ultrasonic waves (waves with frequencies beyond the scope of human hearing) as they travel through materials. The wave propagation within structures yields two key phenomena: alterations in velocity and wave attenuation, stemming from absorption and dispersion mechanisms. As ultrasound waves traverse the material, their energy dissipates, leading to surface reflections. These reflections serve as indicators to identify discontinuities and defects within the material [89].

Among the benefits are the rapid scanning capabilities, the ability to scan intricate shapes, and the potential for real-time monitoring during processes [139].

#### System overview

In GPs, the GW acts as a porous medium, which enhances the transmission and reduces the reflection of the ultrasonic beam. Transducers are strategically placed to emit and capture elastic waves traveling through the material. A transmitter sends out the signal, while a receiver captures it. Important parameters include wavelength, propagation velocity, and time/frequency [89].

**Fig. 32:**
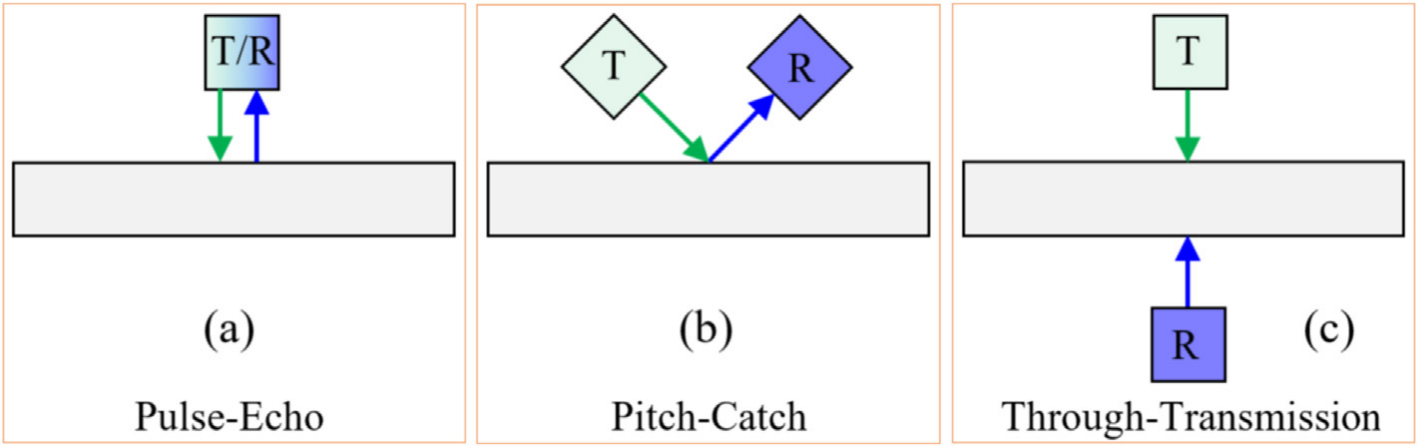
Standard transmission-reception configurations for Pulse-Echo (a), Pitch-Catch (b) and Through-Transmission (c) [89].

Damage between the transmitter and receiver alters the ultrasound signal, allowing for the identification of damaged areas through signal analysis. Fig. 32a and Fig. 32c depict bulk waves used to detect internal flaws, while Fig. 32b shows surface waves used to identify surface anomalies.

#### Applications in conventional and AI-driven Contexts

Arunachalam et al. [139] proposed using the ultrasonic reflection method for non-destructive evaluation to monitor the GW surface continuously. Wear and loading during grinding alter the GW's surface texture, affecting its reflective properties. Changes in the reflection coefficient can be analyzed to assess the condition of the GW's surface.

**Fig. 33:**
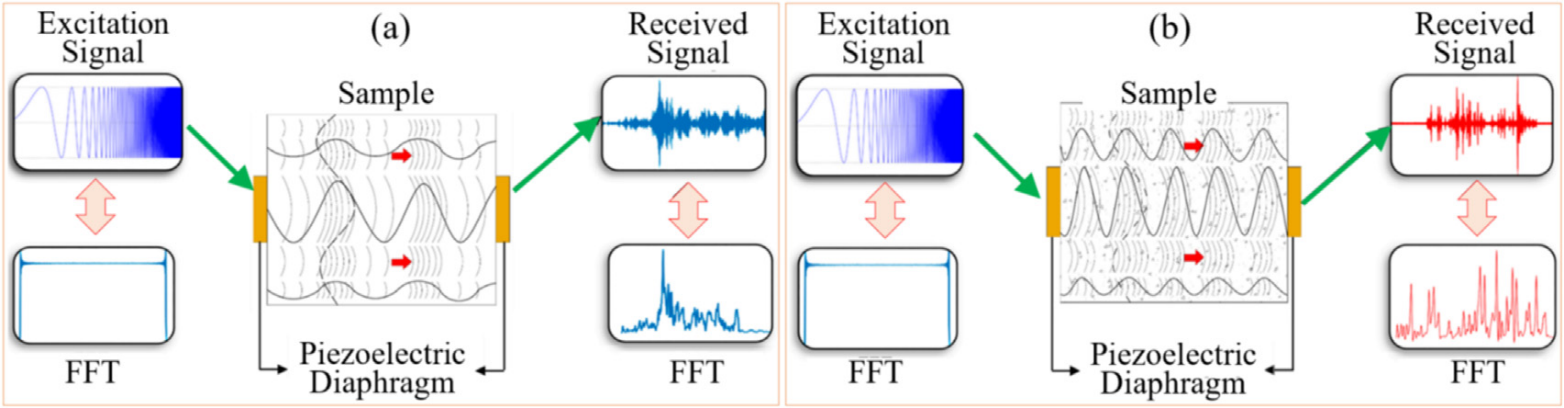
Chirp-Through-Transmission Method: intact structure (a) and compromised structure (b) [89].

Alexandre et al. [89] developed a new ultrasound method using low-cost PZT diaphragms and digital signal processing (DSP) to monitor material removal during grinding. This method, which is an alternative to conventional sensors, uses PZT diaphragms in both active and passive modes. DSP parameters are employed to assess the structural condition. Fig. 33 illustrates the setup and comparison between healthy and damaged structures. Fig. 33a represents a health structure, whereas Fig. 33b depicts a damaged structure.

The emitter, activated by a chirp signal, generates ultrasonic bulk waves that travel through the material. These waves undergo various effects, shaping the emitted signal into a unique signature. Signals from damaged materials differ from those of healthy materials due to structural changes. By comparing these signals using techniques such as spectral analysis, frequency band selection, and statistical measures like RMS and counts, structural alterations can be identified.

#### Summary of the Subsection

In this section, various approaches involving the application of ultrasound sensors for monitoring the GP have been presented. Table 11 provides a summary of the referenced studies.

**Table 11:** Methods of monitoring the GP using ultrasound sensors.

**Grinding Process**	**Year**	**Main application**	**Sensors**	**Reference**
GW	2011	Online monitoring of the GW surface	Conventional ultrasound sensor	[139]
GW	2019	Online monitoring of the material removal	PZT diaphragm	[89]

### Optical and laser sensors

This monitoring method uses a scattered light sensor and a CCD system to capture reflected light from the GW's surface. Light and laser sensors measure parameters such as length, angle, surface characteristics, and irregular coordinates of the GW in real-time during production. Output signals during grinding detect changes in the wear flat area. This technique can measure macroscopic features like radial errors at wheel speeds over 300 m/s, but monitoring micrometric features like wheel wear is limited to speeds up to 20 m/s [140]. Notably, limitations include operating in high temperatures, air-polluted environments, machine vibrations, and susceptibility to environmental influences [141].

#### System overview

**Fig. 34:**
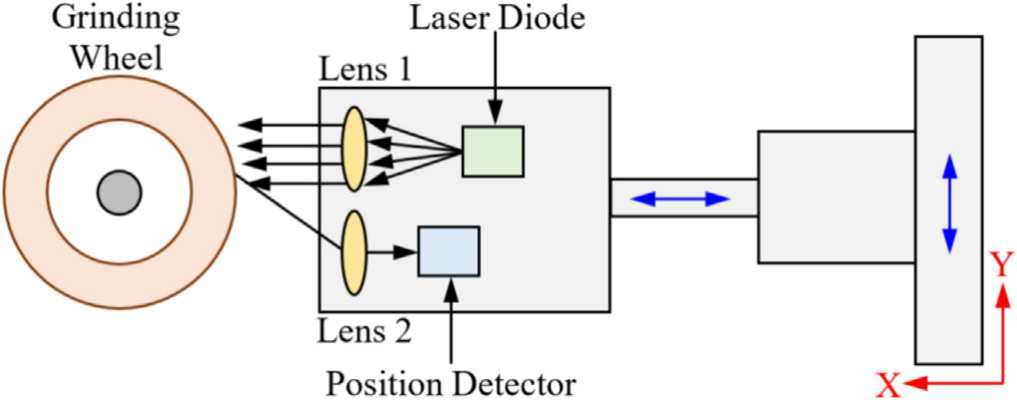
Monitoring of GW surface using laser triangulation [140].

There are two separate systems: one for monitoring the abrasive tool and another for observing the ground surface. Fig. 34 illustrates a GW monitoring system using laser triangulation. This system comprises a laser diode, two lenses, and a position-sensitive detector. The laser diode emits monochromatic light, which is focused onto the GW's surface by the first lens. The second lens then focuses the scattered light onto the detector. The sensor system is mounted on the drive motor, allowing movement perpendicular to the GW along both the X and Y axes [140].

**Fig. 35:**
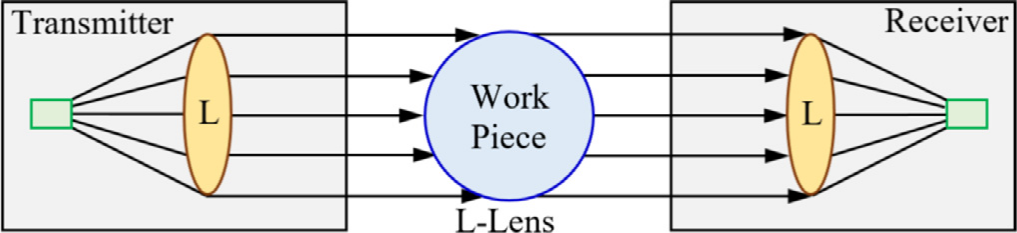
Monitoring the workpiece surface using laser [140].

To monitor the ground surface, surface roughness and waviness are measured by the angular deviation of the incident ray. Fig. 35 illustrates a laser monitoring system that includes a laser diode, rotating mirror, guiding lens, and photodiode to detect dimensional discrepancies in the workpiece [140]. Surface roughness is assessed using structured laser light and image processing of the laser strip [142]. Fu et al. [143] proposed using a laser beam and speckle pattern with 0.2 µm resolution, placing the laser confocal sensor vertically and horizontally to evaluate surface texture features.

#### Applications in conventional and AI-driven Contexts

**Fig. 36:**
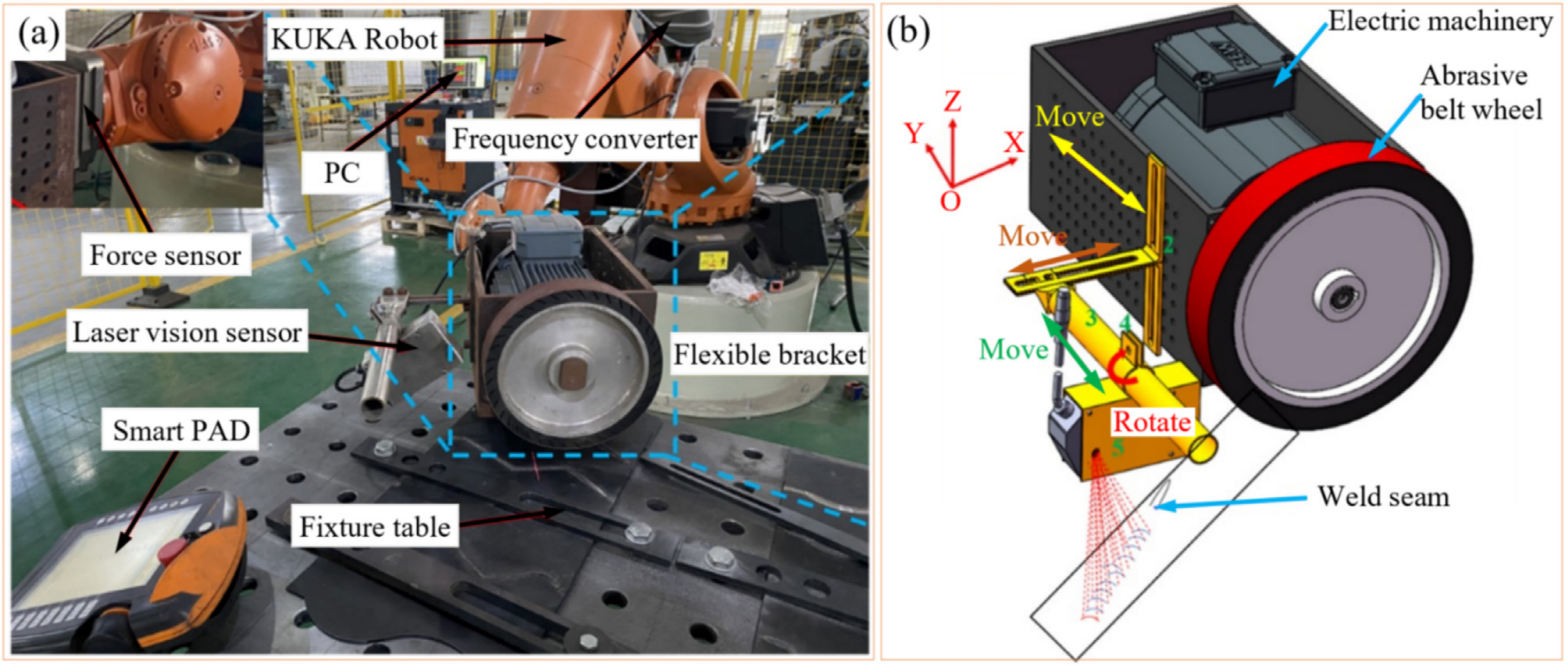
Experimental setup: Hardware components (a) and Bracket design (b) [144].

Ge et al. [144] introduced an adaptive parameter optimization method for robotic grinding of weld seams, utilizing a laser vision sensor and a material removal model. The laser vision sensor's real-time triangulation determines the weld seam's removal depth, addressing issues where the removal depth doesn't match the feed depth due to factors like soft contact and uneven weld height. This method ensures uniform weld seam surfaces. Fig. 36 shows the experimental setup, including hardware components and bracket design.

#### Summary of the Subsection

In this section, various approaches involving the application of optical and laser sensors for monitoring the GP have been presented. Table 12 provides a summary of the referenced studies.

**Table 12:** Methods of monitoring the GP using optics and laser sensors.

**Grinding process**	**Year**	**Main application**	**Sensors**	**Reference**
GW	2023	Adaptive parameter optimization	Laser triangulation vision sensing	[144]
GW	2018	Real-time surface characterization	Confocal Laser	[143]
GW	2022	Determination of the grinding machine's condition	Laser vision sensor based on triangulation	[140]

## Challenges and future trends

One of the main challenges in using advanced sensor systems in grinding operations is integrating them into the demanding environments of industrial machinery. Grinding machines operate at high speeds, generate significant vibrations, and are exposed to cutting fluids and debris. These conditions can compromise the performance and durability of sensors. For instance, vibration and AE sensors often pick up interference from external noise, which requires filtering systems to ensure they work properly. Moreover, grinding operations produce large amounts of data from sensors like force, temperature, and imaging systems. Processing this data in real-time is difficult. Traditional systems struggle with the volume of data, while AI-based systems require powerful computing resources and well-designed algorithms to handle it effectively.

Cost is another barrier to adopting advanced sensor systems, especially for small and medium-sized businesses. AI-driven systems, such as those using machine vision or neural networks, often come with high upfront costs and require ongoing maintenance, making them less accessible to smaller companies. Environmental factors also limit their use. Sensors like IRT and optical sensors are sensitive to high temperatures, cutting fluids, and debris, which can reduce their reliability. Similarly, laser and ultrasonic sensors are affected by vibrations and air quality issues. Another issue is the reliability of some sensors, such as AE and vibration sensors, which can be inconsistent due to external disturbances. Additionally, the lack of standardization across the industry creates compatibility problems, as different machines and systems may not work well together.

Looking ahead, advancements in technology are expected to address many of these challenges. AI and ML are likely to play a bigger role in monitoring tool conditions, planning maintenance, and controlling processes. Techniques like neural networks and SVM could help analyze the complex data generated during grinding operations. Multi-sensor systems that combine data from force, vibration, temperature, AE, and imaging sensors may offer a more complete picture of tool and process conditions, making it easier to detect and address issues during production.

Real-time monitoring and decision-making are also expected to improve as edge computing and faster data processing technologies become more common. These advancements will allow for quicker analysis of sensor data, enabling immediate adjustments during grinding operations. Efforts are also being made to develop sensors that are more durable and affordable, such as PZT sensors and compact imaging systems, which could make these technologies more accessible to smaller businesses. Researchers are also working on improving the resilience of sensors to environmental challenges, such as adapting infrared cameras and optical sensors to perform reliably in fluid-cooled environments.

Wireless and IoT-enabled sensors are expected to become more widely used in grinding operations. These systems will make it easier to monitor processes remotely and share data across different systems, improving overall control and allowing for predictive maintenance. Wireless systems may also simplify installation by reducing the need for extensive wiring. Standardizing sensor systems across the industry could further enhance compatibility and ease of integration. Additionally, sensors are likely to play a key role in optimizing energy use and reducing waste in grinding operations. For example, current and power sensors could help identify inefficiencies in the process, contributing to more sustainable manufacturing practices.

In conclusion, while there are significant challenges in implementing advanced sensor systems in grinding operations, ongoing developments in AI, multi-sensor systems, real-time monitoring, and sensor design are expected to overcome many of these obstacles. These advancements will not only improve the efficiency and reliability of GPs but also support energy conservation and waste reduction, making them more sustainable in the long term.

## Conclusions

In the present study, a comprehensive review of both AI-based and conventional TCM systems in grinding operations was conducted. The integration of various sensors and signal processing techniques was analyzed to highlight their impact on process efficiency, accuracy, and tool life. The following key conclusions can be drawn from this review:

• **In-line Monitoring with Sensors:** Real-time monitoring of grinding operations is effectively achieved using sensors such as vibration, AE, IRT cameras, and dynamometers. For instance, vibration and AE sensors demonstrate up to 90% accuracy in detecting tool wear and system instability, making them invaluable for ensuring consistent machining performance.
• **AI vs. Conventional Systems:** Two primary TCM systems are widely implemented across industries. AI-based systems enable predictive maintenance by forecasting tool performance with over 90% accuracy before machining begins, reducing downtime by up to 25%. In contrast, conventional sensor systems are primarily used during operations to provide real-time data, ensuring immediate corrective actions.
• **Temperature Monitoring:** Temperature measurement is critical for preventing thermal damage to both the tool and the workpiece. While IRT cameras provide a broad temperature distribution view, they often fail to capture the real cutting zone temperature in fluid-cooled environments. TCs, on the other hand, offer precise measurements of the cutting zone, making them more reliable for critical applications.
• **Cutting Force Measurement:** Dynamometers are extensively used to measure cutting forces with over 85% reliability, aiding in machine tool design and optimization. Although AI systems are increasingly preferred for force prediction, the robustness and reliability of dynamometers make them indispensable for accurate force measurements during grinding.
• **Surface Roughness and Morphology:** Evaluating surface roughness and morphology at different stages of machining is crucial for ensuring product quality. Sensors and microscopes are widely used to meet this requirement, with image processing systems achieving sub-micron accuracy in detecting surface defects. High-resolution imaging systems combined with AI algorithms have shown R^2^ as high as 0.96 in predicting surface quality.
• **Acoustic and Vibration Monitoring:** Acoustic sensors play a vital role in grinding operations by enabling the evaluation of machine tool vibrations and GW conditions. These sensors are particularly effective in detecting chatter and ensuring system stability, contributing significantly to process optimization.
• **Image Processing Sensors:** The use of image processing sensors has proven to be highly effective for GW monitoring and defect detection. These sensors, when integrated with AI-driven machine vision systems, offer unparalleled precision in identifying wear and ensuring compatibility with varying machining conditions and parameters.

In summary, this review underscores the importance of integrating advanced TCM systems into grinding operations to enhance process efficiency, reduce defects, and extend tool life. While conventional systems provide reliable real-time monitoring, AI-driven solutions offer predictive capabilities that significantly improve process control and maintenance strategies. The combination of these systems, supported by quantitative metrics such as up to 35% improvement in anomaly detection and over 90% prediction accuracy, represents a transformative step toward smarter, more sustainable manufacturing practices.

## Declaration of competing interest

The authors declare that they have no known competing financial interests or personal relationships that could have appeared to influence the work reported in this paper.
